# Global burden, risk factors, clinicopathological characteristics, molecular biomarkers and outcomes of microsatellite instability-high gastric cancer

**DOI:** 10.18632/aging.205431

**Published:** 2024-01-12

**Authors:** Zhishan Zhang, Jinyuan Huang, Yingying Li, Huimeng Yan, Junxing Xie, Jing Wang, Bin Zhao

**Affiliations:** 1 Quanzhou First Hospital Affiliated to Fujian Medical University, Quanzhou 362000, China; 2 The Second Affiliated Hospital and Yuying Children’s Hospital, Wenzhou Medical University, Wenzhou 325000, China

**Keywords:** microsatellite, gastric cancer, immunotherapy, biomarker

## Abstract

Microsatellite instability-high (MSI-H) has gained considerable interests since it was approved as a tumor-agnostic biomarker in immunotherapy. However, the reported characteristics of MSI-H gastric cancer (GC) are inconsistent due to the biological complexity. Here, we aim to clarify the prevalence, risk factors, clinicopathological/molecular features and outcomes of MSI-H GC though a comprehensive review on 43246 patients from 134 cohorts. Overall, the proportion of MSI-H GC was 14.5% (95% CI, 13.3%-15.8%). Patients with MSI-H GC were less likely to have Epstein-Barr virus infection. High incidences of MSI-H GC were associated with female, older age, lower gastric body, Lauren intestinal histology, WHO tubular and mucinous subtypes, and early disease stage. Additionally, patients with MSI-H GC harbored more *KRAS* mutation, PD-L1 positivity, CD8 overexpression, and higher TMB, but less HER2 positivity and *TP53* mutation. When treated with conventional strategy, the 5-year survival rates in MSI-H patients (70.3%) and MSI-L/MSS patients (43.7%) were significantly different (*p*<0.001). Patients with MSI-H GC derived larger benefit from immunotherapy in term of overall survival (*p_Interaction_*<0.001) and objective response (*p_Interaction_*=0.02). Since the prevalence of MSI-H GC is relatively high and associated with distinct clinicopathological and molecular characteristics, MSI testing should be conducted during standard diagnostical activity. Moreover, giving MSI-H tumors are often diagnosed at early stage and have favorable outcomes, less aggressive treatment strategies may be considered in clinical practice. In summary, this panoramic review may assist in design and/or interpretation of clinical trials, provide references in drug development, and constitute complementary information in drafting the clinical practice guideline.

## INTRODUCTION

With over one million new cases annually, gastric cancer (GC) is the fifth most diagnosed malignancies globally [1]. Moreover, a recent study reported the incidences of GC increased significantly in the younger generation [2]. GC is often associated with unfavorable outcomes, currently it is the third most common cause of cancer-related deaths [1]. As a heterogeneous, complex and multifactorial disease; the inter-patient, intra-patient, and intra-tumoral heterogeneities in GC were crucial barriers in treatment determination [1, 3, 4]. For example, due to the biological differences between tumors from Western and Eastern countries, it is difficult to identify an international accepted standard-of-care therapy [3]. In Asia, surgery plus adjuvant chemotherapy is more frequent choice, while neoadjuvant chemotherapy/radiotherapy is preferred outside of Asia [1, 3, 4]. Now it is generally accepted that the optimal treatment is dependent on the genomic and molecular characteristics of GC. Remarkably, both two well-known proposals, The Cancer Genome Atlas (TCGA) in the US [5] and the Asian Cancer Research Group (ACRG) in Asia [6], established microsatellite instability-high (MSI-H) as a distinct subgroup of GC.

Microsatellites are short and repetitive DNA sequences that distributed randomly through the whole genome. Tumors with MSI prone to a high mutation rate as consequence of a deficient DNA mismatch repair (dMMR) machinery [7]. The high frequencies of gene mutations can induce the presence of neoantigens and a peculiar immunological microenvironment. In fact, MSI/dMMR has emerged as a tumor-agnostic biomarker for immunotherapy since its approval by the US Food and Drug Administration (FDA) in 2017 [7, 8]. However, giving approximately 97% of tumors were microsatellite instability-low/microsatellite instability-stable (MSI-L/MSS) [9], MSI test is not always conducted in real-world practice. Indeed, current guidelines only recommend MSI testing for colorectal and endometrial cancers in Europe [10, 11]. Although the utility of MSI status may help to identify the most effective treatment, the examination of MSI/dMMR during routine diagnostic activity was not recommended in GC partly because there were no reports regarding the prevalence of MSI-H GC worldwide or in various regions. On the other hand, although the association between MSI-H and various clinicopathological factors or the efficacy of treatments have been examined in GC [1, 3, 12], the results were often ambiguous or conflicted due to the biological complexity of GC. Moreover, there are many inconsistent results due to the limited patients enrolled and/or different methods for measuring MSI. Hence, a comprehensive overview of MSI-H GC could have both basic and clinical importance considering no single study has adequate power to draw any solid conclusions.

Here, with accumulating evidence available, we collected 43246 GC patients from 134 studies and carried out a pooled analysis to estimate the overall proportion of patients with MSI-H GC. To evaluate the performances of different MSI testing method, we investigated the prevalence of MSI-H GC examined by polymerase chain reaction (PCR), immunohistochemistry (IHC), and next-generation sequencing (NGS), respectively. Next, we estimated the incidences of five potential epidemiological and risk factors in patients with MSI-H GC and patients with MSI-L/MSS GC, and compared them by calculate the odds ratios (ORs). Similar comparisons were also conducted in nine clinicopathological features and six molecular biomarkers. Moreover, the 5-year survival rates in patients who were treated with conventional strategies were examined. Lastly, we compared the objective response rate (ORR) and overall survival (OS) in MSI-H GC patients and MSI-L/MSS GC patients who were treated with immune checkpoint inhibitors (ICIs). Our panoramic overview on MSI-H gastric cancer may have implications in the personalization of tumor diagnosis, treatment and prognosis.

## MATERIALS AND METHODS

### Search strategy and selection criteria

This study was conducted according to the Preferred Reporting Items for Systematic Reviews and Meta-Analyses statement [13]. A systematic search of Embase, PubMed and Cochrane databases for articles describing prevalence, risk factors, clinicopathological characteristics, molecular biomarkers, and outcomes of MSI-H GC versus MSI-L/MSS GC from inception to December 2022 was carried out. The keywords used were “microsatellite”, “mismatch repair”, “replication error”, and “gastric cancer”. All investigators preformed the initial search independently, carefully reviewed the title and abstract for relevance, and classified the potential articles as excluded, included and uncertain. For uncertain articles, the full-texts were reviewed for the confirmation of eligibility. Any discrepancy was resolved by discussion.

Both inclusion and exclusion criteria were pre-specified. Studies were eligible if they met the following criteria: (1) original articles, including retrospective and prospective cohort studies, on human gastric cancer; (2) published in the English language; (3) available information regarding the proportions, risk factors, clinicopathological characteristics, molecular biomarkers, or outcomes of MSI-H GC. Exclusion criteria were: (1) other studies on this topic, including pre-clinical papers, review articles, early versions of data later published; (2) studies in the pediatric population; (3) data from unpublished studies. When multiple publication from the same databases occurred, we removed the overlapping data and only included the most recent and/or most complete reporting studies.

### Data extraction and analysis

MSI status was determined by PCR, IHC, and NGS. Treatment methods were classified in immunotherapy and conventional therapy. All authors independently extracted study-level information regarding study characteristics (authors, year of publication, country/region of origin, MSI testing method, and number of patients), risk factors (family history of cancer, Epstein-Barr virus [EBV] infection, *H. pylori* infection, smoking, and drinking), clinicopathological characteristics (age, sex, tumor location, Lauren’s classification, WHO classification, TNM stage), molecular biomarkers (HER2, *P53*, *KRAS*, PD-L1, CD8, and tumor mutation burden [TMB]), treatment methods, and clinical outcomes (ORR and OS). Objective response included complete response and partial response determined by tumor assessments from radiological examinations or physical tests. OS was defined as the time period between the date of diagnosis and the date of death by any cause.

The primary outcomes of this study were: (1) the proportion of MSI-H globally; (2) comparison of risk factors, clinicopathological characteristics, and molecular biomarkers between MSI-H GC and MSI-L/MSS GC; and (3) the prognostic and predictive value of MSI-H as a biomarker.

### Quality assessment

Joanna Briggs Institute (JBI) Critical Appraisal Tool was applied for quality assessment [14]. The JBI assessment rates the risk of bias of cohorts according to appropriateness of sample frame, adequacy of sample size, sampling method, methods for identification and measurement of relevant conditions, data analysis, statistical analysis, and response rate adequacy. Potential publication bias was assessed by visual inspection of Begg’s funnel plots, in which the log odds ratios (ORs) were plotted against their standard errors [15].

### Statistical analysis

Statistical heterogeneity for the pooled estimates was evaluated by the Cochrane’s Q statistic and the Higgins *I^2^* measure [16]. The *I^2^* statistic was calculated to assess the extent of inconsistency contributable to the heterogeneity across different studies. The assumption of homogeneity was considered invalid for *I^2^*>25%. When *I^2^* >25%, the effect size was calculated by a random-effects model using the DerSimonian and Laird approach; otherwise, a fixed-model were conducted. The pooled OR and incidences for both prespecified subgroup analysis and post-hoc analyses were calculated using fixed-effects model or random-effects model depending on the heterogeneity of included trials. Analysis of proportions was conducted with a generalized linear mixed model with Clopper-Pearson intervals to estimate the overall proportion and corresponding 95% confidence interval (CI) [17]. Hazard ratio (HR) was applied to compare the survivals in patients treated with ICI-based regimens and chemotherapy. The eligible trials reported their HRs calculated from Cox proportional-hazards models. Two-sided P <0.05 were considered statistically significant. All analysis was conducted by MedCalc 18.2.1 and RStudio 1.3.1093.

## RESULTS

**Figure 1:**
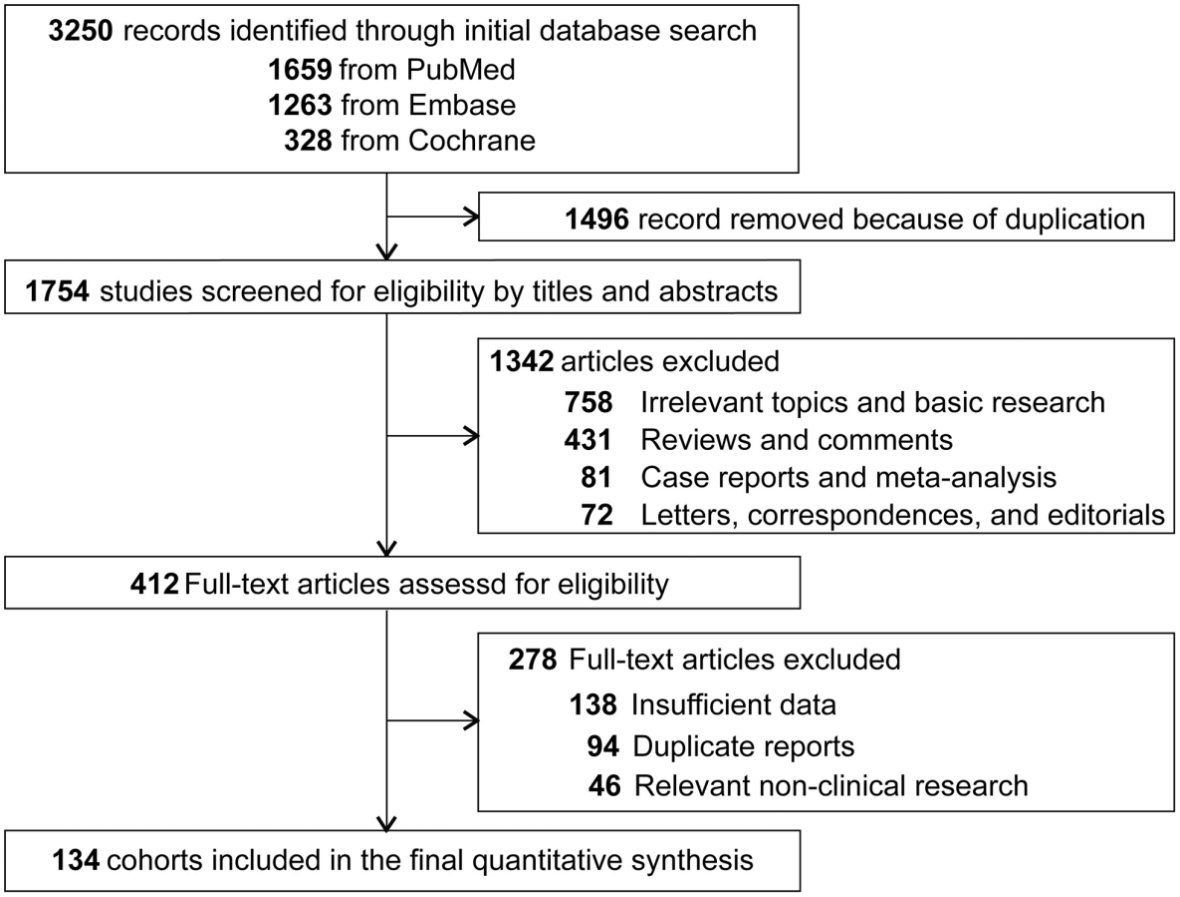
Flowchart diagram of selected cohorts included in this study.

**Figure 2:**
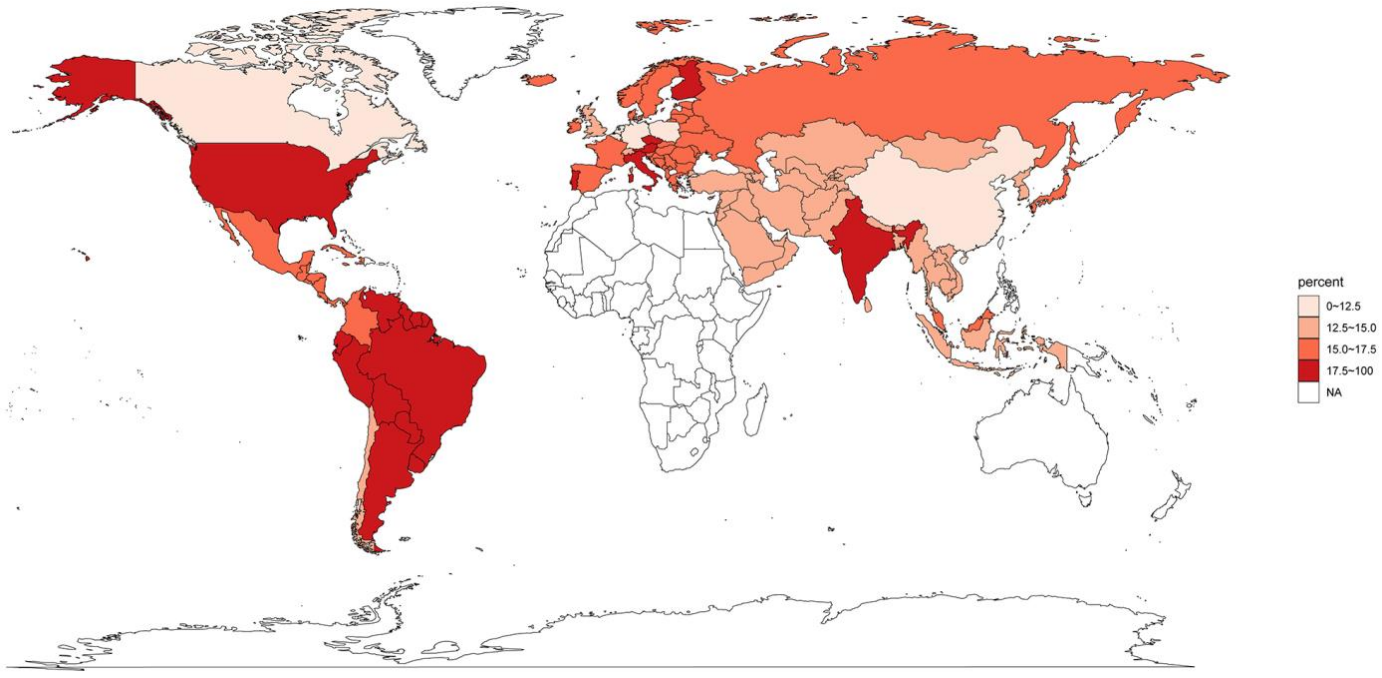
**Proportion of gastric cancer secondary to microsatellite instability high globally.** NA, not available.

The initial search from PubMed, Embase and Cochrane databases yielded 3,250 related papers. After carefully screening and reviewing, 134 cohorts were eligible for the final analysis. A flow chart showing the selection process is presented in Figure 1. All data used for analysis were obtained from published manuscripts. These studies were conducted in Austria, Belgium, Brazil, Canada, China, Chile, Colombia, Czech, Finland, Germany, Hong Kong, India, Italy, Japan, Korea, Malaysia, Netherlands, Poland, Portugal, Spain, Switzerland, Taiwan, UK, and US. Additionally, five international studies [18–24] involved medical centers from other countries including Argentina, Australia, Denmark, Estonia, Greece, Guatemala, Hungary, Ireland, Israel, Latvia, Lithuania, Mexico, New Zealand, Norway, Peru, Puerto Rico, Romania, Russia, Singapore, South Africa, Thailand, and Turkey. The quality of these eligible studies, according to the JBI assessment rates [14], were generally moderate to good (Supplementary Table 1). A total of 43246 patients were enrolled, 4919 with MSI-H GC and 38327 with MSI-L/MSS disease. The overall proportion of patients with MSI-H GC was 14.5% (95% CI, 13.3%-15.8%), it was highest in the South America (21.8%; 95% CI, 17.1%-26.9%), followed by North America (17.9%; 5.9%-34.7%), Europe (16.8%; 14.1%-19.8%), and Asia (13.7%; 12.4%-15.1%) (Figure 2). The global proportion of MSI-H GC remained relatively stable over time periods (14.9%, 95% CI 13.1%-16.9% for before year 2010 vs. 13.2%, 95% CI 10.7%-15.9% for year 2010 and beyond; *p*=0.38).

Different methods were applied to evaluate the MSI status in these eligible studies. PCR was applied in 77 cohorts, IHC in 18 studies, the combination of PCR and IHC in 27 trials, and NGS-based testing in 11 studies. Consist with previous studies [25–27], these different assays showed similar diagnostic performance. Of note, although the prevalence in cohorts that used NGS alone or in combination (13.2%; 95% CI, 9.3%-17.6%) was lower compared with those using PCR and/or IHC (14.9%; 13.7%-16.2%), the difference was not significant (*p*=0.39).

Next, we evaluated the potential epidemiological and risk factors which could increase the incidence of MSI-H GC. As shown in Table 1, MSI-H was independent of familial predisposition (OR, 1.04; 95% CI, 0.64-1.70; *p*=0.87), *H. pylori* infection (0.95; 0.59-1.53; *p*=0.83), smoking (0.55; 0.29-1.05; *p*=0.07), and alcohol consumption (0.89; 0.25-3.17; *p*=0.85). However, patients with MSI-H GC were less likely to have EBV infection than were patients with MSI-L/MSS disease (OR, 0.43; 95% CI, 0.21-0.86; *p*=0.02).

**Table 1:** Characteristics of MSI-H gastric cancer versus MSI-L/MSS gastric cancer.

	**Cohorts, n**	**Patients, n**	**Proportion in MSI-H patients (95% CI)**	**Proportion in MSI-L /MSS patients (95% CI)**	**Odds ratio (95% CI)**	***P*-value**	***I ^2^***
**Risk factors**							
**Family history of cancer**	13	3517					
Yes	13	770	32.0% (21.3-43.8)	32.5% (20.1-46.3)	1.04 (0.64-1.70)	0.87	74
No	13	2143	68.0% (56.2-78.7)	67.5% (53.7-79.9)	0.96 (0.59-1.57)	0.87	74
**Epstein-Barr Virus infection**	18	3820					
Positive	18	311	3.4% (1.3-6.4)	10.3% (7.4-13.6)	0.43 (0.21-0.86)	**0.02**	50
Negative	18	3509	96.6% (93.6-98.7)	89.7% (86.4-92.6)	2.35 (1.17-4.74)	**0.02**	50
***H. pylori* infection**	12	1702					
Positive	12	938	60.0% (40.3-78.2)	56.0% (43.1-68.6)	0.95 (0.59-1.53)	0.83	49
Negative	12	764	40.0% (21.8-59.7)	44.0% (31.4-56.9)	1.05 (0.65-1.70)	0.83	49
**Smoking status**	3	383					
Never/Former smoker	3	178	58.0% (30.9-82.8)	43.7% (27.9-60.3)	1.82 (0.95-3.48)	0.07	31
Current smoker	3	205	42.0% (17.2-69.1)	56.3% (39.7-72.1)	0.55 (0.29-1.05)	0.07	31
**Drinking status**	3	408					
Never/Former drinker	3	216	49.7% (14.2-85.3)	49.9% (8.9-90.9)	1.13 (0.32-4.05)	0.85	72
Current drinker	3	192	50.3% (14.7-85.8)	50.1% (9.1-91.1)	0.89 (0.25-3.17)	0.85	72
**Clinicopathological characteristics**							
**Gender**	86	32366					
Male	86	20867	57.2% (54.3-59.7)	66.8% (64.3-69.2)	0.67 (0.61-0.75)	**<0.001**	43
Female	86	11499	42.8% (40.3-45.4)	33.2% (30.8-35.7)	1.49 (1.34-1.65)	**<0.001**	43
**Age**	28	6433					
<=65 year	28	2754	38.2% (28.4-48.5)	50.1% (42.9-57.3)	0.55 (0.43-0.71)	**<0.001**	51
>65 year	28	3679	61.8% (51.5-71.7)	49.9% (42.6-57.1)	1.80 (1.41-2.32)	**<0.001**	51
**Tumor location**	43	13387					
Cardia tumor	43	2588	12.1% (9.2-15.3)	19.9% (16.5-23.5)	0.55 (0.43-0.71)	**<0.001**	42
Body tumor	43	4037	23.1% (20.2-26.0)	31.8% (29.0-34.7)	0.63 (0.56-0.71)	**<0.001**	5
Antrum tumor	43	6762	63.6% (58.7-68.3)	46.9% (43.0-50.9)	2.17 (1.85-2.53)	**<0.001**	32
**Lauren classification**	62	20007					
Intestinal	62	9706	66.3% (60.7-71.6)	49.8% (47.0-52.6)	2.02 (1.74-2.34)	**<0.001**	45
Diffuse	62	7976	22.4% (17.5-27.7)	40.0% (36.9-43.1)	0.45 (0.39-0.52)	**<0.001**	29
Mixed	62	2325	10.2% (7.2-13.7)	8.5% (6.2-11.2)	1.14 (0.89-1.47)	0.29	51
**WHO classification**	26	8749					
Tubular	26	3823	53.1% (45.2-61.0)	43.7% (37.1-50.4)	1.22 (1.11-1.34)	**<0.001**	50
Poor differentiation	26	4528	42.1% (33.2-51.4)	46.9% (39.3-54.4)	0.79 (0.64-0.97)	**0.02**	40
Signet ring cell	26	303	2.0% (0.8-3.7)	4.1% (1.6-7.5)	0.28 (0.18-0.45)	**<0.001**	4
Mucinous	26	95	1.9% (0.7-3.6)	1.0% (0.4-1.9)	2.02 (1.22-3.35)	**0.01**	32
**Tumor Stage**	57	18208					
Early stage	57	6063	35.2% (29.7-41.0)	30.7% (25.6-36.0)	1.26 (1.07-1.48)	**0.01**	49
Late stage	57	12145	64.8% (59.0-70.3)	69.3% (64.0-74.4)	0.80 (0.68-0.94)	**0.01**	49
**T**	45	22152					
T1/T2	45	8478	37.9% (31.7-44.3)	35.3% (29.9-41.0)	1.07 (0.93-1.25)	0.35	49
T3/T4	45	13674	62.1% (55.7-68.3)	64.7% (59.0-70.1)	0.93 (0.80-1.08)	0.35	49
**N**	65	26025					
N+	65	16509	56.2% (51.4-60.9)	64.5% (60.1-68.8)	0.68 (0.60-0.78)	**<0.001**	42
N-	65	9516	43.8% (39.1-48.6)	35.5% (31.2-39.9)	1.46 (1.29-1.66)	**<0.001**	42
**M**	26	10031					
M0	26	8855	92.7% (88.1-96.2)	87.7% (81.4-92.8)	2.40 (1.88-3.08)	**<0.001**	3
M1	26	1176	7.3% (3.8-11.9)	12.3% (7.2-18.6)	0.42 (0.33-0.53)	**<0.001**	3
**Molecular biomarkers**							
**HER2 expression**	11	4860					
HER2+	11	299	2.6% (1.5-4.3)	8.8% (5.9-12.2)	0.33 (0.19-0.55)	**<0.001**	0
HER2-	11	4561	97.4% (95.8-98.6)	91.2% (87.8-94.1)	3.07 (1.82-5.19)	**<0.001**	0
***TP53* status**	17	2766					
*TP53* mutant	17	1180	24.4% (16.9-32.8)	45.6% (34.2-57.3)	0.39 (0.30-0.50)	**<0.001**	0
*TP53* non-mutant	17	1586	75.6% (67.2-83.1)	54.4% (42.7-65.8)	2.56 (2.00-3.29)	**<0.001**	0
***KRAS* status**	11	1934					
*KRAS* mutant	11	160	20.7% (13.6-28.8)	4.4% (3.2-5.8)	5.56 (3.76-8.23)	**<0.001**	16
*KRAS* non-mutant	11	1774	79.3% (71.2-86.4)	95.6% (94.2-96.8)	0.18 (0.12-0.27)	**<0.001**	16
PD-L1 expression*	17	6129					
PD-L1+	17	2139	61.0% (48.7-72.6)	29.8% (19.9-40.8)	4.04 (2.94-5.56)	**<0.001**	47
PD-L1-	17	3990	39.0% (27.4-51.3)	70.2% (59.2-80.1)	0.25 (0.18-0.34)	**<0.001**	47
**CD8 expression**	6	1677					
High expression	6	1029	73.3% (59.8-84.8)	58.6% (35.7-79.6)	2.34 (1.28-4.27)	**0.006**	63
Low expression	6	648	26.7% (15.2-40.2)	41.4% (20.4-64.3)	0.43 (0.23-0.78)	**0.006**	63
**Tumor mutation burden**	4	1253					
>10 mutations/Mb	4	318	97.9% (91.5-100.0)	13.1% (3.6-27.2)	241.65 (16.52-3535.77)	**<0.001**	78
<=10 mutations/Mb	4	935	2.1% (0.0-8.5)	86.9% (72.8-96.4)	0.004 (0.00-0.06)	**<0.001**	78

*The threshold for PD-L1 positivity/negativity was that PD-L1 stained cell accounting for 1% of tumor cells, or immune and tumor cells evaluated by IHC.

The bold values mean *p*<0.05.

The incidences of MSI-H GC were higher in female (OR, 1.49; 95% CI, 1.34-1.65; *p*<0.001) and older age (>65 years; 1.80; 1.41-2.32; *p*<0.001). MSI-H tumors were more likely found in the lower gastric body (OR, 2.17; 95% CI, 1.85-2.53; *p*<0.001), but not in the upper (0.55; 0.43-0.71; *p*<0.001) and middle body (0.63; 0.56-0.71; *p*<0.001). Compared with MSI-L/MSS GC, more MSI-H tumors were classified as Lauren intestinal subtype (OR, 2.02; 95% CI, 1.74-2.34; *p*<0.001), less as diffuse subtype (0.45; 0.39-0.52; *p*<0.001). According to WHO classification, MSI-H tumors were more likely to identified as tubular subtype (OR, 1.22; 95% CI; 1.11-1.34; *p*<0.001) and mucinous subtype (2.02; 1.22-3.35; *p*=0.01), but less as signet ring cell subtype (0.28; 0.18-0.45; *p*<0.001) and poorly differentiated subtype (0.79; 0.64-0.97; *p*=0.02). Moreover, MSI-H tumors were more often diagnosed at early disease stages (OR, 1.26; 95% CI, 1.07-1.48; *p*=0.01).

MSI-H was associated with higher proportion of *KRAS* mutation (OR, 5.56; 95% CI, 3.76-8.23; *p*<0.001), PD-L1 positivity (4.04; 2.94-5.56; *p*<0.001), CD8 overexpression (2.34; 1.28-4.27; *p*=0.006), and high TMB (TMB>10 mutants/Mb; 241.65; 16.52-3535.77; *p*<0.001), but lower proportion of HER2+ (0.33; 0.19-0.55; *p*<0.001) and *TP53* mutation (0.39; 0.30-0.50; *p*<0.001).

**Figure 3:**
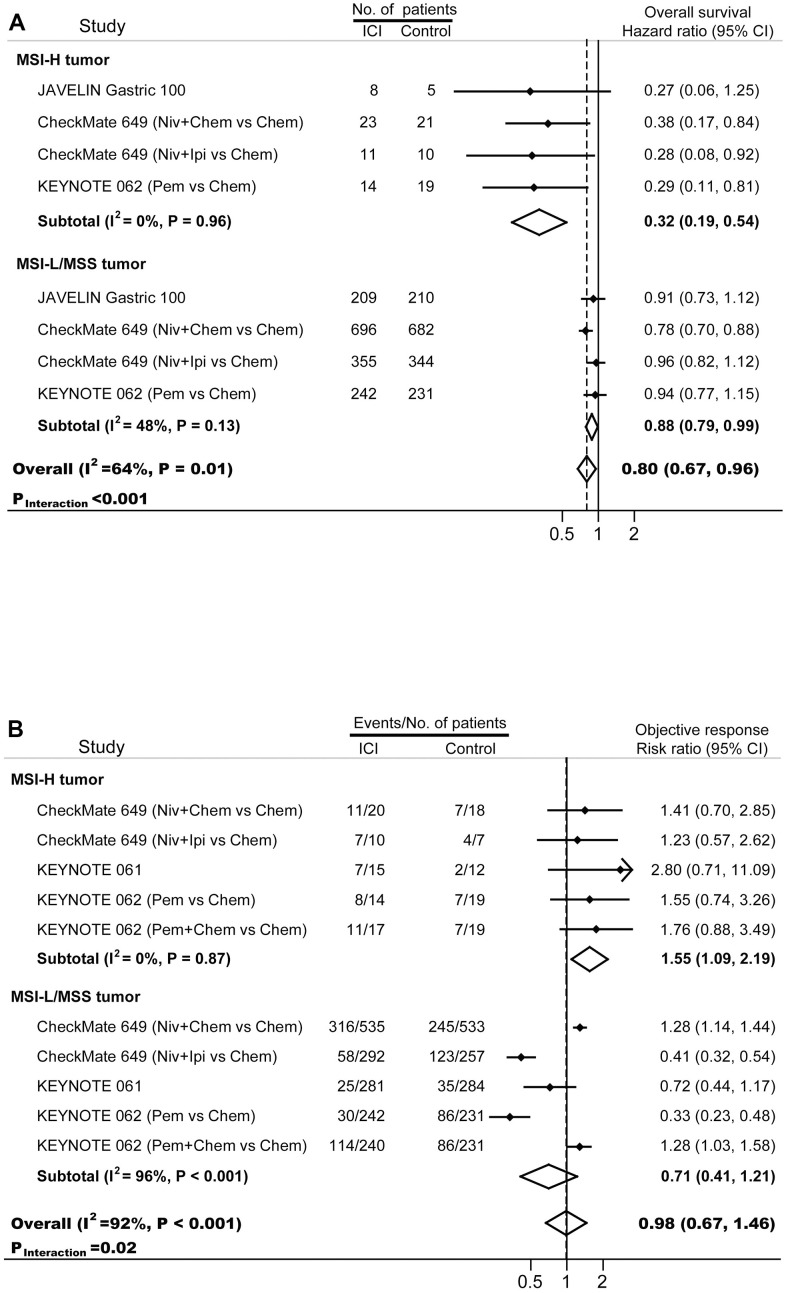
Forest plots of (**A**) hazard ratios for overall survival, and (**B**) risk ratios for objective response in gastric cancer patients treated with immunotherapy. Chem, chemotherapy; ICI, immune checkpoint inhibitor; Ipi, ipilimumab; MSI-H, microsatellite instability-high; MSI-L, microsatellite instability-low; MSS, microsatellite stable; Niv, nivolumab; Pem, pembrolizumab.

The final important aspect to analyze is the clinical outcomes of patients with MSI-H tumors. In 40 cohorts enrolled 17081 patients treated with conventional strategies, 5-year survival rate in MSI-H patients (70.3%; inter quartile range, 57.5%-77.0%) was significant higher compared with that in MSI-L/MSS patients (43.7%, inter quartile range, 36.8%-56.4%; *p*<0.001). Four phase III randomized trials, KEYNOTE-061 [18, 20], KEYNOTE-062 [18, 21], JAVELIN Gastric 100 [22], and CheckMate-649 [19, 23], were included to estimate the activity and efficacy of immunotherapy (Figure 3). Compared with standard treatments, immunotherapy decreased the risk of death by 68% (HR, 0.32; 95% CI, 0.19-0.54) in patients with MSI-H GC and by 12% (HR, 0.88; 95% CI, 0.79-0.99) in patients with MSI-L/MSS GC. The survival outcomes were significantly different between these two subgroups (*p_interaction_*<0.001). More patients with MSI-H GC responded to immunotherapy than to chemotherapy (risk ratio, 1.55; 95% CI, 1.09-2.19; *p*<0.001); whereas similar proportion of MSI-L/MSS GC patients showed responses to immunotherapy and chemotherapy (risk ratio, 0.71; 95% CI, 0.41-1.21). The treatment effect in term of objective response was significantly different between MSI-H GC and MSI-L/MSS GC (*p_interaction_*=0.02).

The Begg’s funnel plots were conducted to evaluate the potential publication bias from every eligible study (Supplementary Figure 1). No significant publication bias was observed.

## DISCUSSION

With published data from 134 cohorts with over 40,000 patients, our pooled analysis first demonstrated that about 14.5% of gastric cancer were secondary to MSI-H globally. The highest proportion of MSI-H GC occurred in South America, and the lowest in East Asia. As the prevalence of MSI-H in colorectal tumor, the most frequently studied tumor types, was about 14.2% in the US [28], we recommended MSI testing should be the first-line analysis during standard diagnostical activity. Moreover, we compared the incidences of five epidemiological and risk factors, nine clinicopathological features and six molecular biomarkers in patients with MSI-H GC and patients with MSI-L/MSS GC, confirmed the distinct characteristics of MSI-H GC. Lastly, our data revealed that MSI-H was a predictive biomarker for better survivals in both conventional treatments and immunotherapy. Since MSI-H tumors were often diagnosed at early stage and had favorable outcomes, less aggressive treatment strategies might be considered in clinical practice. Our panoramic review on MSI-H GC may assist in design and/or interpretation of clinical trials, provide references in drug development, and constitute complementary information in drafting the clinical practice guideline.

Gastric cancer was a malignancy strongly associated with the geographical background. It was well-established that the incidences, clinicopathological characteristics, treatment strategies, and outcomes showed great geographic variations [1, 2]. Due to the important role of MSI-H as a biomarker in cancer immunotherapy, the determination of the prevalence of MSI-H GC from different countries/regions appeared to be an essential prerequisite for worldwide clinical development of ICI-based treatment. Here, our study presented the first global estimated of gastric cancer secondary to MSI-H disease. As expected, the prevalence of MSI-H GC also differed across countries. Interestingly, although the highest incidence of GC was observed among Asiatic population and lowest in Europe and Northern America [2], our data revealed that, compared with Western countries, the proportion of MSI-H GC was significantly lower in East Asia. Surprisingly, the frequency of MSI-H GC was highest in Hispanics/Latinos. Considering the limited enrollment of Latinos in clinical trials, the surveillance strategies for MSI status needed to improve in these patient populations. Currently, the exact explanations for this disproportionate distribution of MSI-H GC are unclear. It seemed that the combination of genetic predisposition, dietary habits and other environmental factors played major roles. For example, in an Italian population highly susceptible to GC, MSI-H was believed to cause the genetic alterations in non-invasive neoplasia [29]. Additionally, the role of dietary risk factors in MSI-H GC was evaluated in a population-based study [30]. They discovered that MSI-H GC was associated with a specific diet pattern, frequent consumption of fresh vegetables and fruits can significantly reduce the risk of MSI-H GC, while high consumption of meat paste, red meat, and nitrite increased the risk. Accordingly, the so-called western-style food habit, which was often referred to refined compounds, red meat, and processed meat, might be a potential reason for the upregulated proportion of MSI-H GC among Western countries. Another hypothesis is that GC is more likely diagnosed at a younger age in East Asia [31, 32] and MSI-H is often associated with older age. Age might be an un-neglected factor in explain the different prevalence of MSI-H GC between Asia and Western countries.

Consist with previous reports [12, 33], our study demonstrated that MSI-H GC occurred more often in older patients. The tumors in young patients and old patients showed different clinicopathological and molecular characteristics. GC in old patients were usually located in the lower body, with relatively low metastasis, and were present in about 10% synchronous GC [34]. These features were often observed in MSI-H GC. In contrast, tumors in young patients were located in middle body, with high metastasis, and occurring in 3% synchronous GC [34]. Moreover, epigenetic changes were involved in the development of GC in old patients [35]. Age related gene methylation could increase the chances of development of malignant neoplasms as CpG island methylation played a key role in the in activation of many genes [35]. For example, it was reported that the methylation of *hMLH1* and its loss of expression were greatly upregulated in aged patient [36], which could significantly increase the possibility of microsatellite instability.

The Epstein-Barr Virus infects 90% of the population worldwide and can directly cause EBV-associated GC [37]. This specific subtype of GC represented a distinct etiologic entity which was associated with mutations in *PIK3A*, hypermethylation of CDKN2A, amplification of *JAK2*, proximal location, male gender, and poorly differentiated histology characteristically with lymphocytic infiltration [38]. Interestingly, none of these features were dominated in MSI-H GC. Indeed, experiments revealed a mutually elite pattern between the presence of EBV positivity and MSI-H that are independent of each other [5, 37], suggesting EBV-associated GC and MSI-H GC involved different molecular pathways during cancer development. Indeed, our analysis here revealed that almost all MSI-H GC were EBV negative (~97%). The EBV positive and MSI-H tumors might belong to the special subset of GC with increased number of lymphocytes [39]. Interestingly, high proportion PD-L1expression was found in both EBV-associated GC [38] and MSI-H GC, and more patients responded to ICI-based immunotherapy compared with other subtypes [3, 37].

Through examining a series of clinicopathological features and molecular biomarkers, our study revealed that a specific genetic profile and distinct clinicopathological characteristics were associated with MSI-H GC. *TP53* was the most commonly mutated gene in tumors and associated with poor outcomes in cancers [40]. In the TCGA analysis, although *TP53* mutations were often observed in chromosomally unstable tumors, they were rarely found in MSI-H cases [5, 41]. It might because *MSH2* and *TP53* genes protected the genome integrity by different pathways [42]. Consist with previous findings in colorectal cancer [43], our data here demonstrated that, compared with MSI-L/MSS GC, the proportion of *TP53* mutations decreased significantly in MSI-H tumors. It was suggested that only a special restricted pattern of P53 expression was preferentially associated with MSI-H phenotype [44]. Interestingly, *TP53* can exert anti-tumor immune activities by increasing antigen presentation, reducing PD-L1 expression [45, 46], and *TP53* dysfunction could repress immunogenic activity by decrease the expression levels of almost all immune-related gene pathways [41]. This might indicate that *TP53* and its associated genes could be a potential biomarker in cancer immunotherapy. Currently, HER2 was the only biomarker which was routinely examined and widely used for targeted therapy in GC [1, 3]. It is a subtype included in the chromosomal instability (CIN) category according to TCGA classification [5], and MSS/TP53 inactive category based on the ACRG classification [6]. HER2+ GC was more commonly associated with proximal location, metastasis, male gender, advanced tumor stage at diagnosis and poor prognosis [47–49]. However, most MSI-H tumors did not have these features. Indeed, our data showed that less than 3% of all MSI-H GC were HER2 positive. It seemed that HER2 positive and MSI-H tumors demonstrated a mutually negative association. This suggested that HER2 and MSI could modulate the tumor microenvironment and the immunologic response in different pathways [50]. It was reported there was a synergistic effect of HER2-targeted therapy and immunotherapy [51], which might explain the recent accelerated approval of pembrolizumab in combination with trastuzumab plus chemotherapy for patients with HER2+ gastric cancer by FDA [52].

In MSI-H tumors, due to the massive production of abnormal tumor-specific neoantigens which could activate recruitment of lymphocytes, a robust correlation between tumor infiltrating lymphocytes (TILs) and MSI was confirmed [53], and a permissive inflamed microenvironment was established [54]. This strong activation of the immune system was one of the explanations for the favorable prognosis and the low rates of metastasis in MSI GC [55]. As expected, our results showed that MSI-H GC had higher PD-L1 expression, CD8+ TIL, and TMB. Numerous evidences revealed the superior efficacy of immunotherapy-based regimens compared with conventional treatment in MSI-H/dMMR patients, even in trials with unfavorable results in the overall population [18, 21]. In the present meta-analysis, ICI-based regimens significantly improved overall survivals and objective response rates in the subgroup of patients with MSI-H GC. Furthermore, the interaction between the outcomes and MSI status remained significant, suggesting that, even if some patients with MSI-L/MSS GC may benefit from immunotherapy (mainly those with PD-L1 positive and/or high tumor mutation burden), the efficacy and activity of immunotherapy in the MSI-H arm is higher compared with the overall MSI-L/MSS counterpart. In fact, because the prognostic value of PD-L1 expression was controversial [56], it was suggested that the combined assessment of MSI status and PD-L1 expression were more powerful than PD-L1 alone in guiding patients’ stratification for immunotherapy [57].

In the past several years, many studies evaluated the clinical relevance of the MSI status as a positive predictor in GC patients [24, 58]. It had been argued that it was due to the correlation between MSI-H with relatively early TNM stage at diagnosis and Lauren intestinal histotype [12, 59]. Interestingly, MSI-H GC was often associated with longer survivals even in patients with advanced disease since these tumors had a lower prevalence of lymph node metastases and a lower ability to invade serosal layers [60]. Because of the prognostic relevance, MSI status should be considered in the therapeutic decision-making to avoid potential excessive medical treatment. For example, it was known that peri-operative chemotherapy is guideline-endorsed treatment for GC [1, 3]. However, in MAGIC trial [61], patients with MSI-H tumor exhibited unfavorable survivals in the chemotherapy plus surgery arm. In CLASSIC study [62], patients with MSI-H GC experience no benefit from chemotherapy in term of disease-free survival. These results confirmed that lack of survival benefit from peri-operative chemotherapy, and hence transforming the clinical practice of operable MSI-H GC.

Our study has several clinical implications. First of all, although the utility of MSI status in clinical practice may help to identify the most effective treatment, the MSI test in gastric cancer is not always conducted in real-world. For example, the diagnosis of MSI status is required only in patients with colorectal and endometrial cancer in Europe [10, 11]. Giving the relatively high prevalence of MSI-H GC in western countries, the examination of MSI/dMMR should be recommended during routine diagnostic activity in gastric cancer. Second, MSI-H GC is associated with relatively early stage at diagnosis, Lauren intestinal histotype, lower prevalence of metastases, and hence favorable outcomes. Additionally, peri-operative chemotherapy exhibited poorly prognosis in patients with MSI-H GC despite it is endorsed by guidelines in gastric cancer treatment [61]. Accordingly, in clinical practice, less aggressive treatment strategies may be considered for patients with MSI-H GC. Furthermore, since patients with MSI-H GC were regarded as a special immune-sensitive papulation, immunotherapy should be routinely available for those with advanced MSI-H GC. Another potential use of this study is in the economic analysis. With the MSI status testing, different treatment strategy will be carried out to achieve the best clinical benefit. Considering ICIs are among the most expensive agents in the world, the financial consequences are significant for patients, their families, and the whole society.

To our knowledge, the present study provides the most comprehensive analysis of the existing literature regarding the panoramic landscape of MSI-H GC to date. However, our study is not without limitations. First, there are very few studies from South-East Asia, South Asia, and Eastern Mediterranean region, and sample size are relatively small. Accordingly, it is cautious to properly interpret the prevalence of MSI-H GC from these regions and more data are needed. Additionally, we cannot extract any information regarding MSI-H GC from Africa and Oceania region. Second, there are substantial heterogeneities in some comparisons, which might arise from the large number of enrolled patients included in the pooled analysis. We clarify the potential sources of the heterogeneities by performing meta-regression and subgroup analysis where appropriate. Third, we conduct the current study at the trial level, no features at individual levels are investigated. It may reduce the reliability in the association between MSI-H and variables in specific subgroup analysis. Fourth, some included information, such as HER2 and CD8 expression status, are reported from various medical centers by different investigators. These data are potentially associated with subjectivity. Our study is subject to any errors and bias from the original researchers.

## CONCLUSIONS

In summary, this study conducts a systematic overview of the global burden, risk factors, clinicopathological characteristics, molecular biomarkers, and clinical outcomes of MSI-H gastric cancer. We provide high-level evidence showing that 15% GC patients have MSI-H disease, which is associated with a specific genetic profile and distinct clinicopathological characteristics. Accordingly, MSI/dMMR should be determined as the first-line analysis during GC standard diagnostic activity. Moreover, giving MSI-H tumors are often diagnosed at relatively early stage and have favorable outcomes, less aggressive treatment strategies may be considered in clinical practice. For patients with advanced MSI-H GC, immunotherapy should be routinely available. Further investigations are needed to better understand the significant etiological factors associated with MSI-H GC.

## Supplementary Material

Supplementary Figure 1

Supplementary Table 1
