# Supplementary material for: Global burden, risk factors, clinicopathological characteristics, molecular biomarkers and outcomes of microsatellite instability-high gastric cancer
Source: Aging (Albany NY). 2024 Jan 12;16(1):948–63. doi: 10.18632/aging.205431 (PMC10817383; doi:10.18632/aging.205431)
Supplement: Supplementary Figure 1 [file aging-16-205431-s001.pdf]

SUPPLEMENTARY FIGURES

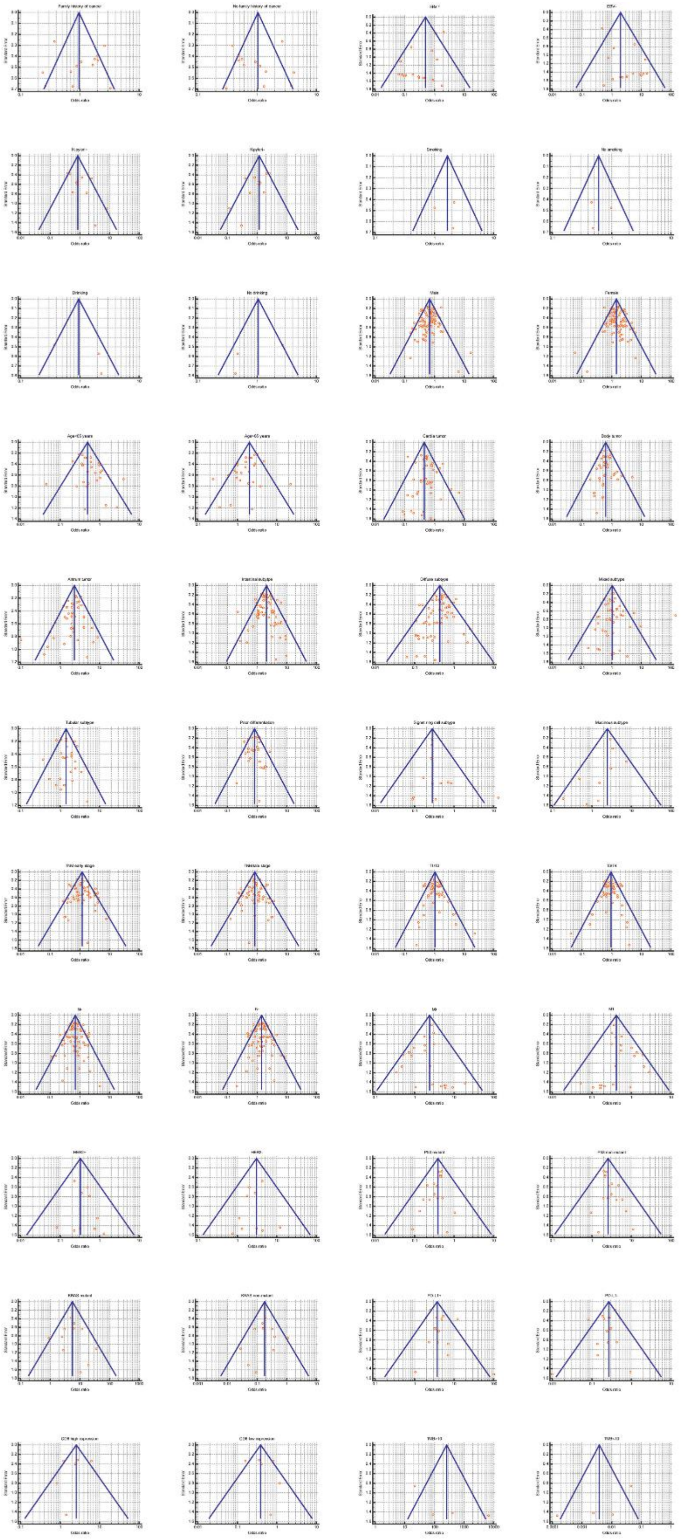

Supplementary Figure 1. Begg's funnel plot of eligible trials to evaluate the potential publication bias.
