# Supplementary material for: Global burden, risk factors, clinicopathological characteristics, molecular biomarkers and outcomes of microsatellite instability-high gastric cancer
Source: Aging (Albany NY). 2024 Jan 12;16(1):948–63. doi: 10.18632/aging.205431 (PMC10817383; doi:10.18632/aging.205431)
Supplement: Supplementary Table 1 [file aging-16-205431-s002.docx]

**Supplementary Table 1.** Summary of eligible studies.

| **Author, year** | **Country/Region** | **No. of patients** | **MSI-H testing method** | **Sample size (MSI-H)** | **Sample size (MSI-L/MSS)** | **Age (mean, years)** | **Gender**  **Male (%)** | **Quality assessment** |
| --- | --- | --- | --- | --- | --- | --- | --- | --- |
| Abe,2012^1^ | Japan | 793 | IHC | 136 | 657 | 65 | 68.5 | 7 |
| Ahn,2017^2^; Kim,2013^3^ | Korea | 414 | PCR, IHC | 23 | 391 | 62 | 64.5 | 8 |
| An,2012^4^; Kim,2011^5^; Jahng,2012^6^ | Korea | 1990 | PCR | 170 | 1820 | 59 | 66.9 | 8 |
| An,2020^7^ | Korea | 790 | PCR, IHC | 64 | 726 | 57 | NR | 7 |
| Bacani,2005^8^ | Canada | 139 | PCR, IHC | 7 | 132 | 42 | 62.6 | 7 |
| Beghelli,2006^9^ | Italy | 510 | PCR, IHC | 83 | 427 | 64 | 62.6 | 7 |
| Bermudez,2021^10^ | Spain | 142 | IHC | 23 | 119 | 65 | 62.7 | 8 |
| Bevilacqua,2000^11^ | Brazil | 42 | PCR | 8 | 34 | 62 | 50.0 | 7 |
| Biesma,2022^12^ | Netherlands | 901 | PCR, IHC | 74 | 827 | 64 | 62.6 | 9 |
| Buonsanti,1997^13^ | Italy | 98 | PCR | 14 | 84 | NR | NR | 7 |
| Cai,2020^14^ | China | 271 | PCR | 28 | 243 | 65 | 67.2 | 8 |
| Cai,2021^15^; Cai,2020^16^ | China | 1757 | PCR, IHC | 185 | 1572 | 64 | 67.8 | 8 |
| Carvalho,2004^17^ | Netherlands | 62 | PCR, IHC | 0 | 62 | 33 | NR | 7 |
| Chakraborty,2021^18^ | India | 80 | PCR | 32 | 48 | 60 | 53.8 | 8 |
| Chang,2002^19^ | Korea | 129 | PCR | 20 | 109 | 55 | NR | 7 |
| Chang,2018^20^; Cho,2018^21^ | Korea | 204 | PCR | 79 | 125 | NR | NR | 7 |
| Chao,2021^22^;Shitara,2018^23^; Shitara,2020^24^ | Multi-country | 1614 | PCR | 84 | 1530 | 62 | 71.1 | 9 |
| Chiaravalli,2006^25^ | Italy | 96 | IHC | 35 | 61 | 66 | 59.4 | 7 |
| Choi,2000^26^ | Korea | 118 | PCR | 20 | 98 | 59 | 67.0 | 7 |
| Choi,2015^27^ | Korea | 623 | PCR | 68 | 555 | 61 | 68.2 | 8 |
| Choi,2019^28^ | Korea | 592 | PCR | 40 | 552 | 57 | 70.1 | 7 |
| Choi,2020^29^ | Korea | 514 | PCR, IHC | 53 | 461 | 65 | 67.5 | 8 |
| Chong,1994^30^ | Japan | 76 | PCR | 25 | 51 | NR | NR | 6 |
| Chung,1999^31^ | Korea | 51 | PCR | 11 | 40 | NR | NR | 7 |
| Cordova-Delgado,2019^32^ | Chile | 48 | NGS | 7 | 41 | NR | 72.9 | 7 |
| Czopek,2002^33^ | Poland | 76 | PCR | 11 | 65 | 63 | NR | 8 |
| Dai,2020^34^ | China | 89 | PCR | 18 | 71 | NR | 75.3 | 7 |
| Daun,2021^35^ | Switzerland | 115 | NGS | 20 | 95 | 74 | 64.4 | 8 |
| De Meulder,2022^36^ | Belgium | 114 | PCR, IHC | 11 | 103 | 67 | 63.6 | 7 |
| D'Errico,2009^37^ | Italy | 131 | PCR | 20 | 111 | NR | NR | 7 |
| Di Bartolomeo,2020^38^ | Italy | 256 | PCR | 24 | 232 | 62 | NR | 8 |
| Dislich,2020^39^ | Switzerland | 415 | IHC | 49 | 366 | 71 | 61.9 | 7 |
| Falchetti,2008^40^ | Italy | 159 | PCR, IHC | 27 | 132 | NR | 64.8 | 7 |
| Fang,1999^41^; Fang,2001^42^ | China | 68 | PCR | 8 | 60 | NR | 73.6 | 7 |
| Fang,2019^43^ | Taiwan | 356 | PCR | 34 | 322 | NR | NR | 7 |
| Fang,2020^44^; Huang,2020^45^ | Taiwan | 360 | PCR, IHC | 59 | 301 | NR | 68.1 | 7 |
| Fukushima,2022^46^ | Japan | 115 | PCR | 48 | 67 | NR | 28.7 | 7 |
| Furlan,2002^47^ | Italy | 300 | PCR, IHC | 55 | 245 | NR | NR | 7 |
| Giampieri,2017^48^ | Italy | 103 | IHC | 15 | 88 | NR | 68.9 | 7 |
| Grundei,2000^49^ | Germany | 37 | PCR | 2 | 35 | 54 | NR | 7 |
| Guan,2021^50^ | China | 890 | PCR, IHC | 196 | 694 | 58 | 64.0 | 8 |
| Haag,2019^51^ | Germany | 101 | PCR, IHC | 9 | 92 | 61 | NR | 8 |
| Halling,1999^52^ | US and Italy | 117 | PCR, IHC | 10 | 107 | 67 | 72.7 | 8 |
| Haron,2019^53^ | Malaysia | 60 | PCR, NGS | 10 | 50 | NR | 63.3 | 7 |
| Hasegawa,2022^54^ | Japan | 31 | PCR | 7 | 24 | 69 | NR | 7 |
| Hasuo,2007^55^ | Japan | 110 | PCR, IHC | 9 | 101 | NR | 67.3 | 7 |
| Hayden,1997^56^ | UK | 101 | PCR | 21 | 80 | 70 | 63.4 | 7 |
| Herz,2022^57^ | Germany | 583 | PCR, IHC | 53 | 530 | 63 | NR | 7 |
| Hewitt,2018^58^ | UK, Japan | 1094 | IHC | 113 | 981 | NR | 67.6 | 7 |
| Hirata,2007^59^; Suzuki,1999^60^; Yamamoto,1999^61^ | Japan | 205 | PCR | 30 | 175 | NR | NR | 7 |
| Hiyama,2004^62^ | Japan | 98 | PCR | 12 | 86 | NR | 71.4 | 7 |
| Huang,2010^63^ | China | 276 | PCR | 23 | 253 | 60 | 68.9 | 7 |
| Huang,2019^64^ | Taiwan | 1248 | IHC | 116 | 1132 | 66 | 62.3 | 8 |
| Huang,2021^65^ | China | 192 | NGS | 13 | 179 | 62 | 67.8 | 7 |
| Jee,1997^66^ | Korea | 77 | PCR | 17 | 60 | 59 | 54.6 | 7 |
| Jiao,2004^67^ | Japan | 76 | PCR | 8 | 68 | NR | NR | 7 |
| Karpinska-Kaczmarczyk,2016^68^ | Poland | 107 | IHC | 6 | 101 | 65 | 62.6 | 8 |
| Kim,2003^69^ | Korea | 79 | PCR | 36 | 43 | NR | 64.6 | 7 |
| Kim,2003^70^ | Korea | 116 | PCR | 16 | 100 | 61 | 59.5 | 7 |
| Kim,2010^71^ | Korea | 128 | PCR | 14 | 114 | 60 | 68.8 | 7 |
| Kim,2015^72^; Shin,2019^73^ | Korea | 1276 | PCR, IHC | 105 | 1171 | 58 | 68.3 | 7 |
| Kim,2016^74^ | Korea | 434 | PCR | 41 | 393 | 62 | 68.0 | 7 |
| Kim,2018^75^ | Korea | 61 | PCR, IHC | 7 | 54 | 57 | NR | 9 |
| Kim,2019^76^ | Korea | 297 | IHC | 32 | 265 | 63 | NR | 7 |
| Kim,2020^77^ | Korea | 359 | PCR | 41 | 318 | 60 | 65.7 | 8 |
| Kim,2021^78^ | Korea | 185 | IHC | 19 | 166 | 59 | NR | 7 |
| Kleo,2022^79^ | Germany | 29 | PCR | 4 | 25 | 64 | 62.1 | 7 |
| Kohlruss,2021^80^; Kohlruss,2019^81^ | Germany | 717 | PCR | 67 | 650 | 65 | 73.8 | 8 |
| Lee,2001^82^ | China | 109 | PCR | 24 | 85 | 58 | 67.9 | 7 |
| Lee,2002^83^; Chang,2003^84^ | Korea | 327 | PCR, IHC | 31 | 296 | 55 | 67.6 | 7 |
| Lee,2020^85^ | Korea | 136 | PCR | 16 | 120 | 57 | NR | 7 |
| Leite,2011^86^ | Portugal, Italy | 410 | PCR, IHC | 95 | 315 | 67 | 67.0 | 7 |
| Li,2005^87^ | China | 46 | PCR | 12 | 34 | NR | NR | 7 |
| Li,2021^88^ | China | 377 | PCR | 13 | 364 | 59 | 72.4 | 7 |
| Li,2021^89^ | China | 1568 | PCR | 128 | 1440 | 60 | 71.7 | 7 |
| Lim,2014^90^; Koh,2019^91^; Park,2021^92^ | Korea | 1107 | PCR | 86 | 1021 | 59 | NR | 8 |
| Lin,1995^93^; Wu,1997^94^ | Taiwan | 59 | PCR | 20 | 39 | NR | 60.0 | 7 |
| Liu,2005^95^ | China | 36 | PCR | 7 | 29 | 59 | 69.4 | 8 |
| Liu,2018^96^ | US | 383 | NGS | 73 | 310 | 65 | 65.8 | 9 |
| Liu,2020^97^; Cristescu,2015^98^ | Korea | 300 | IHC, NGS | 67 | 233 | NR | 66.3 | 7 |
| Ma,2009^99^ | China | 90 | PCR | 13 | 77 | NR | 74.4 | 7 |
| Ma,2016^100^ | US | 44 | IHC | 16 | 28 | 73 | 56.9 | 7 |
| Mizoshita,2005^101^ | Japan | 70 | PCR, IHC | 13 | 57 | NR | NR | 6 |
| Moehler,2020^102^ | Multi-country | 432 | PCR | 13 | 419 | 62 | 66.3 | 9 |
| Oh,2021^103^ | Korea | 838 | PCR | 100 | 738 | 58 | 63.0 | 7 |
| Oki,2009^104^ | Japan | 240 | PCR | 22 | 218 | 64 | 60.3 | 7 |
| Oki,2009^105^; Sakurai,2007^106^ | Japan | 56 | PCR | 4 | 52 | 62 | 60.7 | 7 |
| Ottini,1997^107^ | Italy | 108 | PCR, IHC | 33 | 75 | NR | 64.8 | 7 |
| Palli,2001^108^ | Italy | 126 | PCR | 43 | 83 | NR | 65.1 | 7 |
| Park,2010^109^ | Korea | 191 | PCR | 28 | 163 | 57 | NR | 7 |
| Park,2017^110^ | Korea | 52 | PCR | 8 | 44 | 66 | 63.5 | 8 |
| Pascua,2015^111^ | Spain | 75 | PCR | 14 | 61 | NR | NR | 7 |
| Pereira,2018^112^ | Brazil | 222 | IHC | 60 | 162 | 62 | 58.1 | 7 |
| Pietrantonio,2019^113^ | Multi-country | 1556 | PCR | 121 | 1435 | 59 | 70.1 | 9 |
| Pinto,2000^114^; Santos,1996^115^ | Portugal | 57 | PCR | 28 | 29 | 63 | 49.1 | 7 |
| Polom,2018^116^ | Italy, German | 176 | PCR | 14 | 162 | 66 | 56.8 | 7 |
| Polom,2019^117^; Polom,2016^118^; Pedrazzani,2009^119^; Marrelli,2016^120^; Corso,2009^121^; Corso,2011^122^ | Italy | 595 | PCR | 121 | 474 | 75 | 60.0 | 9 |
| Pretzsch,2022^123^ | Germany | 189 | IHC | 19 | 170 | 65 | 63.0 | 7 |
| Quaas,2021^124^ | Germany | 582 | PCR, IHC | 44 | 538 | NR | 66.3 | 7 |
| Ramos,2021^125^ | Brazil | 287 | IHC | 58 | 229 | 61 | 70.0 | 8 |
| Renault,1996^126^ | Italy | 40 | PCR | 13 | 27 | NR | NR | 7 |
| Rugge,2005^127^ | Italy | 55 | PCR, IHC | 5 | 50 | NR | NR | 7 |
| Schlintl,2022^128^ | Austria | 39 | PCR, IHC, NGS | 8 | 31 | 58 | NR | 7 |
| Schneider,2000^129^ | US, Colombia, Chile, Korea | 169 | PCR | 29 | 140 | 67 | 67.0 | 7 |
| Seo,2009^130^ | Korea | 328 | PCR, IHC | 27 | 301 | NR | 72.3 | 7 |
| Sepulveda,1999^131^ | US, Colombia, Korea | 68 | PCR | 16 | 52 | NR | NR | 7 |
| Seruca,1995^132^ | Portugal | 34 | PCR | 11 | 23 | 62 | 58.8 | 7 |
| Shen,2017^133^ | China | 202 | PCR | 15 | 187 | NR | 68.8 | 7 |
| Shirai,2006^134^ | Japan | 181 | PCR | 16 | 165 | 62 | 72.9 | 8 |
| Shitara,2022^135^; Janjigian,2021^136^ | Multi-country | 1421 | NR | 44 | 1377 | 62 | 69.6 | 9 |
| Stanek,2022^137^ | Czech | 40 | IHC | 16 | 24 | NR | NR | 6 |
| Sugimoto,2021^138^; Sugai,2018^139^; Sugimoto,2016^140^ | Japan | 330 | PCR | 45 | 285 | 75 | 73.9 | 8 |
| Takahashi,2002^141^ | Japan | 65 | PCR | 14 | 51 | 66 | NR | 7 |
| Tanabe,2021^142^ | Japan | 94 | PCR, IHC | 10 | 84 | 73 | 76.6 | 8 |
| Theuer,2002^143^ | Japan, US | 38 | PCR | 11 | 27 | 69 | 52.6 | 7 |
| Vauhkonen,2005^144^ | Finland | 37 | PCR | 7 | 30 | NR | 59.5 | 7 |
| Velho,2005^145^ | Portugal | 47 | PCR | 26 | 21 | NR | NR | 7 |
| Wang,2021^146^ | China | 205 | IHC | 46 | 159 | NR | NR | 7 |
| Wu,2004^147^ | Japan | 62 | PCR | 14 | 48 | NR | NR | 6 |
| Wu,2020^148^; Fang,2012^149^ | Taiwan | 433 | PCR | 40 | 393 | 67 | 71.0 | 7 |
| Xiao,2006^150^ | China | 50 | PCR | 11 | 39 | NR | NR | 6 |
| Yamada,2002^151^ | Japan | 96 | PCR | 14 | 82 | 65 | 69.8 | 7 |
| Yamashita,2013^152^ | Japan | 123 | PCR | 12 | 111 | NR | NR | 6 |
| Yamazaki,2006^153^ | Japan | 219 | PCR | 16 | 203 | NR | NR | 7 |
| Yoshida,2022^154^ | Japan | 379 | PCR | 24 | 355 | NR | NR | 7 |
| Yu,2021^155^ | China | 529 | NGS | 60 | 469 | NR | NR | 7 |
| Yu,2022^156^ | Taiwan | 52 | IHC | 8 | 44 | 66 | NR | 7 |
| Yuza,2021^157^ | Japan | 124 | NGS | 13 | 111 | 68 | NR | 7 |
| Zaky,2008^158^ | Japan | 95 | PCR | 31 | 64 | 67 | 82.1 | 7 |
| Zhang,2018^159^ | China | 567 | IHC | 57 | 510 | NR | 68.4 | 7 |
| Zhang,2021^160^;Wang,2022^161^ | China | 2031 | IHC, NGS | 140 | 1891 | NR | 28.1 | 7 |
| Zhang,2021^162^ | China | 150 | NGS | 8 | 142 | 61 | NR | 8 |
| Zhao,2004^163^; Leung,1999^164^ | China | 94 | PCR | 21 | 73 | NR | 70.9 | 7 |
| Zhao,2015^165^ | China | 210 | PCR | 22 | 188 | 64 | 66.2 | 7 |

IHC, immunohistochemistry; MSI-H, microsatellite instability-high; MSI-L, microsatellite instability-low; MSS, microsatellite stable; NGS, next generation sequencing; NR, not reported; PCR, polymerase chain reaction.

References

1. Abe H, Maeda D, Hino R, et al. ARID1A expression loss in gastric cancer: pathway-dependent roles with and without Epstein-Barr virus infection and microsatellite instability. *Virchows Archiv : an international journal of pathology.* 2012;461(4):367-377.

2. Ahn S, Lee SJ, Kim Y, et al. High-throughput Protein and mRNA Expression-based Classification of Gastric Cancers Can Identify Clinically Distinct Subtypes, Concordant With Recent Molecular Classifications. *The American journal of surgical pathology.* 2017;41(1):106-115.

3. Kim JY, Shin NR, Kim A, et al. Microsatellite instability status in gastric cancer: a reappraisal of its clinical significance and relationship with mucin phenotypes. *Korean journal of pathology.* 2013;47(1):28-35.

4. An JY, Kim H, Cheong JH, Hyung WJ, Kim H, Noh SH. Microsatellite instability in sporadic gastric cancer: its prognostic role and guidance for 5-FU based chemotherapy after R0 resection. *Int J Cancer.* 2012;131(2):505-511.

5. Kim H, An JY, Noh SH, Shin SK, Lee YC, Kim H. High microsatellite instability predicts good prognosis in intestinal-type gastric cancers. *Journal of gastroenterology and hepatology.* 2011;26(3):585-592.

6. Jahng J, Youn YH, Kim KH, et al. Endoscopic and clinicopathologic characteristics of early gastric cancer with high microsatellite instability. *World journal of gastroenterology.* 2012;18(27):3571-3577.

7. An JY, Choi YY, Lee J, et al. A Multi-cohort Study of the Prognostic Significance of Microsatellite Instability or Mismatch Repair Status after Recurrence of Resectable Gastric Cancer. *Cancer Res Treat.* 2020;52(4):1153-1161.

8. Bacani J, Zwingerman R, Di Nicola N, et al. Tumor microsatellite instability in early onset gastric cancer. *The Journal of molecular diagnostics : JMD.* 2005;7(4):465-477.

9. Beghelli S, de Manzoni G, Barbi S, et al. Microsatellite instability in gastric cancer is associated with better prognosis in only stage II cancers. *Surgery.* 2006;139(3):347-356.

10. Bermúdez A, Arranz-Salas I, Mercado S, et al. Her2-Positive and Microsatellite Instability Status in Gastric Cancer-Clinicopathological Implications. *Diagnostics (Basel, Switzerland).* 2021;11(6).

11. Bevilacqua RA, Simpson AJ. Methylation of the hMLH1 promoter but no hMLH1 mutations in sporadic gastric carcinomas with high-level microsatellite instability. *Int J Cancer.* 2000;87(2):200-203.

12. Biesma HD, Soeratram TTD, Sikorska K, et al. Response to neoadjuvant chemotherapy and survival in molecular subtypes of resectable gastric cancer: a post hoc analysis of the D1/D2 and CRITICS trials. *Gastric cancer : official journal of the International Gastric Cancer Association and the Japanese Gastric Cancer Association.* 2022;25(3):640-651.

13. Buonsanti G, Calistri D, Padovan L, et al. Microsatellite instability in intestinal- and diffuse-type gastric carcinoma. *The Journal of pathology.* 1997;182(2):167-173.

14. Cai L, Sun Y, Wang K, et al. The Better Survival of MSI Subtype Is Associated With the Oxidative Stress Related Pathways in Gastric Cancer. *Frontiers in oncology.* 2020;10:1269.

15. Cai Z, Song H, Fingerhut A, et al. A greater lymph node yield is required during pathological examination in microsatellite instability-high gastric cancer. *BMC cancer.* 2021;21(1):319.

16. Cai Z, Rui W, Li S, et al. Microsatellite Status Affects Tumor Response and Survival in Patients Undergoing Neoadjuvant Chemotherapy for Clinical Stage III Gastric Cancer. *Frontiers in oncology.* 2020;10:614785.

17. Carvalho R, Milne AN, van Rees BP, et al. Early-onset gastric carcinomas display molecular characteristics distinct from gastric carcinomas occurring at a later age. *The Journal of pathology.* 2004;204(1):75-83.

18. Chakraborty P, Ghatak S, Chenkual S, et al. Panel of significant risk factors predicts early stage gastric cancer and indication of poor prognostic association with pathogens and microsatellite stability. *Genes and environment : the official journal of the Japanese Environmental Mutagen Society.* 2021;43(1):3.

19. Chang MS, Kim HS, Kim CW, Kim YI, Lan Lee B, Kim WH. Epstein-Barr virus, p53 protein, and microsatellite instability in the adenoma-carcinoma sequence of the stomach. *Human pathology.* 2002;33(4):415-420.

20. Chang YH, Heo YJ, Cho J, Song SY, Lee J, Kim KM. Computational measurement of tumor immune microenvironment in gastric adenocarcinomas. *Scientific reports.* 2018;8(1):13887.

21. Cho J, Chang YH, Heo YJ, et al. Four distinct immune microenvironment subtypes in gastric adenocarcinoma with special reference to microsatellite instability. *ESMO open.* 2018;3(3):e000326.

22. Chao J, Fuchs CS, Shitara K, et al. Assessment of Pembrolizumab Therapy for the Treatment of Microsatellite Instability-High Gastric or Gastroesophageal Junction Cancer Among Patients in the KEYNOTE-059, KEYNOTE-061, and KEYNOTE-062 Clinical Trials. *JAMA oncology.* 2021;7(6):895-902.

23. Shitara K, Ozguroglu M, Bang YJ, et al. Pembrolizumab versus paclitaxel for previously treated, advanced gastric or gastro-oesophageal junction cancer (KEYNOTE-061): a randomised, open-label, controlled, phase 3 trial. *Lancet (London, England).* 2018;392(10142):123-133.

24. Shitara K, Van Cutsem E, Bang YJ, et al. Efficacy and Safety of Pembrolizumab or Pembrolizumab Plus Chemotherapy vs Chemotherapy Alone for Patients With First-line, Advanced Gastric Cancer: The KEYNOTE-062 Phase 3 Randomized Clinical Trial. *JAMA oncology.* 2020.

25. Chiaravalli AM, Feltri M, Bertolini V, et al. Intratumour T cells, their activation status and survival in gastric carcinomas characterised for microsatellite instability and Epstein-Barr virus infection. *Virchows Archiv : an international journal of pathology.* 2006;448(3):344-353.

26. Choi SW, Choi JR, Chung YJ, Kim KM, Rhyu MG. Prognostic implications of microsatellite genotypes in gastric carcinoma. *Int J Cancer.* 2000;89(4):378-383.

27. Choi J, Nam SK, Park DJ, et al. Correlation between microsatellite instability-high phenotype and occult lymph node metastasis in gastric carcinoma. *APMIS : acta pathologica, microbiologica, et immunologica Scandinavica.* 2015;123(3):215-222.

28. Choi YY, Kim H, Shin SJ, et al. Microsatellite Instability and Programmed Cell Death-Ligand 1 Expression in Stage II/III Gastric Cancer: Post Hoc Analysis of the CLASSIC Randomized Controlled study. *Annals of surgery.* 2019;270(2):309-316.

29. Choi E, Chang MS, Byeon SJ, et al. Prognostic perspectives of PD-L1 combined with tumor-infiltrating lymphocytes, Epstein-Barr virus, and microsatellite instability in gastric carcinomas. *Diagnostic pathology.* 2020;15(1):69.

30. Chong JM, Fukayama M, Hayashi Y, et al. Microsatellite instability in the progression of gastric carcinoma. *Cancer research.* 1994;54(17):4595-4597.

31. Chung YJ, Kim KM, Choi JR, Choi SW, Rhyu MG. Relationship between intratumor histological heterogeneity and genetic abnormalities in gastric carcinoma with microsatellite instability. *Int J Cancer.* 1999;82(6):782-788.

32. Cordova-Delgado M, Pinto MP, Retamal IN, et al. High Proportion of Potential Candidates for Immunotherapy in a Chilean Cohort of Gastric Cancer Patients: Results of the FORCE1 Study. *Cancers (Basel).* 2019;11(9).

33. Czopek J, Bialas M, Rudzki Z, et al. The relationship between gastric cancer cells circulating in the blood and microsatellite instability positive gastric carcinomas. *Alimentary pharmacology & therapeutics.* 2002;16 Suppl 2:128-136.

34. Dai D, Zhao X, Li X, et al. Association Between the Microsatellite Instability Status and the Efficacy of Postoperative Adjuvant Chemoradiotherapy in Patients With Gastric Cancer. *Frontiers in oncology.* 2019;9:1452.

35. Daun T, Nienhold R, Paasinen-Sohns A, et al. Combined Simplified Molecular Classification of Gastric Adenocarcinoma, Enhanced by Lymph Node Status: An Integrative Approach. *Cancers (Basel).* 2021;13(15).

36. De Meulder S, Sagaert X, Brems H, et al. Prevalence of microsatellite instable and Epstein-Barr Virus-driven gastroesophageal cancer in a large Belgian cohort. *Acta gastro-enterologica Belgica.* 2022;85(1):1-5.

37. D'Errico M, de Rinaldis E, Blasi MF, et al. Genome-wide expression profile of sporadic gastric cancers with microsatellite instability. *European journal of cancer (Oxford, England : 1990).* 2009;45(3):461-469.

38. Di Bartolomeo M, Morano F, Raimondi A, et al. Prognostic and Predictive Value of Microsatellite Instability, Inflammatory Reaction and PD-L1 in Gastric Cancer Patients Treated with Either Adjuvant 5-FU/LV or Sequential FOLFIRI Followed by Cisplatin and Docetaxel: A Translational Analysis from the ITACA-S Trial. *The oncologist.* 2020;25(3):e460-e468.

39. Dislich B, Blaser N, Berger MD, Gloor B, Langer R. Preservation of Epstein-Barr virus status and mismatch repair protein status along the metastatic course of gastric cancer. *Histopathology.* 2020;76(5):740-747.

40. Falchetti M, Saieva C, Lupi R, et al. Gastric cancer with high-level microsatellite instability: target gene mutations, clinicopathologic features, and long-term survival. *Human pathology.* 2008;39(6):925-932.

41. Fang DC, Jass JR, Wang DX, Zhou XD, Luo YH, Young J. Infrequent loss of heterozygosity of APC/MCC and DCC genes in gastric cancer showing DNA microsatellite instability. *Journal of clinical pathology.* 1999;52(7):504-508.

42. Fang DC, Yang SM, Zhou XD, Wang DX, Luo YH. Telomere erosion is independent of microsatellite instability but related to loss of heterozygosity in gastric cancer. *World journal of gastroenterology.* 2001;7(4):522-526.

43. Fang WL, Huang KH, Chang SC, et al. Comparison of the Clinicopathological Characteristics and Genetic Alterations Between Patients with Gastric Cancer with or Without Helicobacter pylori Infection. *The oncologist.* 2019;24(9):e845-e853.

44. Fang WL, Chen MH, Huang KH, et al. The Clinicopathological Features and Genetic Mutations in Gastric Cancer Patients According to EMAST and MSI Status. *Cancers (Basel).* 2020;12(3).

45. Huang KH, Chen MH, Fang WL, et al. The Clinicopathological Characteristics And Genetic Alterations of Signet-ring Cell Carcinoma in Gastric Cancer. *Cancers (Basel).* 2020;12(8).

46. Fukushima M, Fukui H, Watari J, et al. Gastric Xanthelasma, Microsatellite Instability and Methylation of Tumor Suppressor Genes in the Gastric Mucosa: Correlation and Comparison as a Predictive Marker for the Development of Synchronous/Metachronous Gastric Cancer. *Journal of clinical medicine.* 2021;11(1).

47. Furlan D, Casati B, Cerutti R, et al. Genetic progression in sporadic endometrial and gastrointestinal cancers with high microsatellite instability. *The Journal of pathology.* 2002;197(5):603-609.

48. Giampieri R, Maccaroni E, Mandolesi A, et al. Mismatch repair deficiency may affect clinical outcome through immune response activation in metastatic gastric cancer patients receiving first-line chemotherapy. *Gastric cancer : official journal of the International Gastric Cancer Association and the Japanese Gastric Cancer Association.* 2017;20(1):156-163.

49. Grundei T, Vogelsang H, Ott K, et al. Loss of heterozygosity and microsatellite instability as predictive markers for neoadjuvant treatment in gastric carcinoma. *Clinical cancer research : an official journal of the American Association for Cancer Research.* 2000;6(12):4782-4788.

50. Guan WL, Ma Y, Cui YH, et al. The Impact of Mismatch Repair Status on Prognosis of Patients With Gastric Cancer: A Multicenter Analysis. *Frontiers in oncology.* 2021;11:712760.

51. Haag GM, Czink E, Ahadova A, et al. Prognostic significance of microsatellite-instability in gastric and gastroesophageal junction cancer patients undergoing neoadjuvant chemotherapy. *Int J Cancer.* 2019;144(7):1697-1703.

52. Halling KC, Harper J, Moskaluk CA, et al. Origin of microsatellite instability in gastric cancer. *The American journal of pathology.* 1999;155(1):205-211.

53. Haron NH, Mohamad Hanif EA, Abdul Manaf MR, et al. Microsatellite Instability and Altered Expressions of MLH1 and MSH2 in Gastric Cancer. *Asian Pacific journal of cancer prevention : APJCP.* 2019;20(2):509-517.

54. Hasegawa H, Shitara K, Takiguchi S, et al. A multicenter, open-label, single-arm phase I trial of neoadjuvant nivolumab monotherapy for resectable gastric cancer. *Gastric cancer : official journal of the International Gastric Cancer Association and the Japanese Gastric Cancer Association.* 2022;25(3):619-628.

55. Hasuo T, Semba S, Li D, et al. Assessment of microsatellite instability status for the prediction of metachronous recurrence after initial endoscopic submucosal dissection for early gastric cancer. *British journal of cancer.* 2007;96(1):89-94.

56. Hayden JD, Cawkwell L, Quirke P, et al. Prognostic significance of microsatellite instability in patients with gastric carcinoma. *European journal of cancer (Oxford, England : 1990).* 1997;33(14):2342-2346.

57. Herz AL, Wisser S, Kohlruss M, et al. Elevated microsatellite instability at selected tetranucleotide (EMAST) repeats in gastric cancer: a distinct microsatellite instability type with potential clinical impact? *The journal of pathology Clinical research.* 2022;8(3):233-244.

58. Hewitt LC, Inam IZ, Saito Y, et al. Epstein-Barr virus and mismatch repair deficiency status differ between oesophageal and gastric cancer: A large multi-centre study. *European journal of cancer (Oxford, England : 1990).* 2018;94:104-114.

59. Hirata T, Yamamoto H, Taniguchi H, et al. Characterization of the immune escape phenotype of human gastric cancers with and without high-frequency microsatellite instability. *The Journal of pathology.* 2007;211(5):516-523.

60. Suzuki H, Itoh F, Toyota M, et al. Distinct methylation pattern and microsatellite instability in sporadic gastric cancer. *Int J Cancer.* 1999;83(3):309-313.

61. Yamamoto H, Itoh F, Fukushima H, Hinoda Y, Imai K. Overexpression of cyclooxygenase-2 protein is less frequent in gastric cancers with microsatellite instability. *Int J Cancer.* 1999;84(4):400-403.

62. Hiyama T, Tanaka S, Yoshihara M, et al. Chromosomal and microsatellite instability in sporadic gastric cancer. *Journal of gastroenterology and hepatology.* 2004;19(7):756-760.

63. Huang YQ, Yuan Y, Ge WT, Hu HG, Zhang SZ, Zheng S. Comparative features of colorectal and gastric cancers with microsatellite instability in Chinese patients. *Journal of Zhejiang University Science B.* 2010;11(9):647-653.

64. Huang SC, Ng KF, Yeh TS, et al. Subtraction of Epstein-Barr virus and microsatellite instability genotypes from the Lauren histotypes: Combined molecular and histologic subtyping with clinicopathological and prognostic significance validated in a cohort of 1,248 cases. *Int J Cancer.* 2019;145(12):3218-3230.

65. Huang H, Wang Z, Li Y, Zhao Q, Niu Z. Amplification of the human epidermal growth factor receptor 2 (HER2) gene is associated with a microsatellite stable status in Chinese gastric cancer patients. *Journal of gastrointestinal oncology.* 2021;12(2):377-387.

66. Jee MS, Koo C, Kim MH, et al. Microsatellite instability in Korean patients with gastric adenocarcinoma. *The Korean journal of internal medicine.* 1997;12(2):144-154.

67. Jiao YF, Sugai T, Habano W, Suzuki M, Takagane A, Nakamura S. Analysis of microsatellite alterations in gastric carcinoma using the crypt isolation technique. *The Journal of pathology.* 2004;204(2):200-207.

68. Karpińska-Kaczmarczyk K, Lewandowska M, Ławniczak M, Białek A, Urasińska E. Expression of Mismatch Repair Proteins in Early and Advanced Gastric Cancer in Poland. *Medical science monitor : international medical journal of experimental and clinical research.* 2016;22:2886-2892.

69. Kim H, Kim YH, Kim SE, Kim NG, Noh SH, Kim H. Concerted promoter hypermethylation of hMLH1, p16INK4A, and E-cadherin in gastric carcinomas with microsatellite instability. *The Journal of pathology.* 2003;200(1):23-31.

70. Kim KM, Kwon MS, Hong SJ, et al. Genetic classification of intestinal-type and diffuse-type gastric cancers based on chromosomal loss and microsatellite instability. *Virchows Archiv : an international journal of pathology.* 2003;443(4):491-500.

71. Kim SH, Ahn BK, Nam YS, Pyo JY, Oh YH, Lee KH. Microsatellite instability is associated with the clinicopathologic features of gastric cancer in sporadic gastric cancer patients. *Journal of gastric cancer.* 2010;10(4):149-154.

72. Kim SY, Choi YY, An JY, et al. The benefit of microsatellite instability is attenuated by chemotherapy in stage II and stage III gastric cancer: Results from a large cohort with subgroup analyses. *Int J Cancer.* 2015;137(4):819-825.

73. Shin SJ, Kim SY, Choi YY, et al. Mismatch Repair Status of Gastric Cancer and Its Association with the Local and Systemic Immune Response. *The oncologist.* 2019;24(9):e835-e844.

74. Kim YB, Lee SY, Kim JH, et al. Microsatellite Instability of Gastric and Colorectal Cancers as a Predictor of Synchronous Gastric or Colorectal Neoplasms. *Gut and liver.* 2016;10(2):220-227.

75. Kim ST, Cristescu R, Bass AJ, et al. Comprehensive molecular characterization of clinical responses to PD-1 inhibition in metastatic gastric cancer. *Nature medicine.* 2018;24(9):1449-1458.

76. Kim JY, Kim WG, Kwon CH, Park DY. Differences in immune contextures among different molecular subtypes of gastric cancer and their prognostic impact. *Gastric cancer : official journal of the International Gastric Cancer Association and the Japanese Gastric Cancer Association.* 2019;22(6):1164-1175.

77. Kim JW, Cho SY, Chae J, et al. Adjuvant Chemotherapy in Microsatellite Instability-High Gastric Cancer. *Cancer Res Treat.* 2020;52(4):1178-1187.

78. Kim N, Yu JI, Lim DH, et al. Prognostic Impact of Sarcopenia and Radiotherapy in Patients With Advanced Gastric Cancer Treated With Anti-PD-1 Antibody. *Frontiers in immunology.* 2021;12:701668.

79. Kleo K, Jovanovic VM, Arndold A, et al. Response prediction in patients with gastric and esophagogastric adenocarcinoma under neoadjuvant chemotherapy using targeted gene expression analysis and next-generation sequencing in pre-therapeutic biopsies. *Journal of cancer research and clinical oncology.* 2022.

80. Kohlruss M, Ott K, Grosser B, et al. Sexual Difference Matters: Females with High Microsatellite Instability Show Increased Survival after Neoadjuvant Chemotherapy in Gastric Cancer. *Cancers (Basel).* 2021;13(5).

81. Kohlruss M, Grosser B, Krenauer M, et al. Prognostic implication of molecular subtypes and response to neoadjuvant chemotherapy in 760 gastric carcinomas: role of Epstein-Barr virus infection and high- and low-microsatellite instability. *The journal of pathology Clinical research.* 2019;5(4):227-239.

82. Lee TL, Leung WK, Lau JY, et al. Inverse association between cyclooxygenase-2 overexpression and microsatellite instability in gastric cancer. *Cancer letters.* 2001;168(2):133-140.

83. Lee HS, Choi SI, Lee HK, et al. Distinct clinical features and outcomes of gastric cancers with microsatellite instability. *Modern pathology : an official journal of the United States and Canadian Academy of Pathology, Inc.* 2002;15(6):632-640.

84. Chang MS, Lee HS, Kim HS, et al. Epstein-Barr virus and microsatellite instability in gastric carcinogenesis. *The Journal of pathology.* 2003;199(4):447-452.

85. Lee J, Chung SJ, Choi JM, Han YM, Kim JS. Clinicopathologic Characteristics and Long-Term Outcome of Gastric Cancer Patients with Family History: Seven-Year Follow-Up Study for Korean Health Check-Up Subjects. *Gastroenterology research and practice.* 2020;2020:4028136.

86. Leite M, Corso G, Sousa S, et al. MSI phenotype and MMR alterations in familial and sporadic gastric cancer. *Int J Cancer.* 2011;128(7):1606-1613.

87. Li JH, Shi XZ, Lv S, Liu M, Xu GW. Effect of Helicobacter pylori infection on p53 expression of gastric mucosa and adenocarcinoma with microsatellite instability. *World journal of gastroenterology.* 2005;11(28):4363-4366.

88. Li X, Zhang L, Wang C, et al. Microsatellite instability in Chinese gastric cancer and its correlation with clinical characteristics. *Journal of gastrointestinal oncology.* 2021;12(6):2719-2727.

89. Li Z, Wang Y, Ying X, et al. Prognostic and predictive value of mismatch repair deficiency in gastric and gastroesophageal junction adenocarcinoma patients receiving neoadjuvant or adjuvant chemotherapy. *Journal of surgical oncology.* 2021;124(8):1356-1364.

90. Lim JH, Lee DH, Shin CM, et al. Clinicopathological features and surgical safety of gastric cancer in elderly patients. *Journal of Korean medical science.* 2014;29(12):1639-1645.

91. Koh J, Nam SK, Roh H, et al. Somatic mutational profiles of stage II and III gastric cancer according to tumor microenvironment immune type. *Genes, chromosomes & cancer.* 2019;58(1):12-22.

92. Park Y, Seo AN, Koh J, et al. Expression of the immune checkpoint receptors PD-1, LAG3, and TIM3 in the immune context of stage II and III gastric cancer by using single and chromogenic multiplex immunohistochemistry. *Oncoimmunology.* 2021;10(1):1954761.

93. Lin JT, Wu MS, Shun CT, et al. Microsatellite instability in gastric carcinoma with special references to histopathology and cancer stages. *European journal of cancer (Oxford, England : 1990).* 1995;31a(11):1879-1882.

94. Wu MS, Sheu JC, Shun CT, et al. Infrequent hMSH2 mutations in sporadic gastric adenocarcinoma with microsatellite instability. *Cancer letters.* 1997;112(2):161-166.

95. Liu P, Zhang XY, Shao Y, Zhang DF. Microsatellite instability in gastric cancer and pre-cancerous lesions. *World journal of gastroenterology.* 2005;11(31):4904-4907.

96. Liu J, Lichtenberg T, Hoadley KA, et al. An Integrated TCGA Pan-Cancer Clinical Data Resource to Drive High-Quality Survival Outcome Analytics. *Cell.* 2018;173(2):400-416.e411.

97. Liu X, Choi MG, Kim K, et al. High PD-L1 expression in gastric cancer (GC) patients and correlation with molecular features. *Pathology, research and practice.* 2020;216(4):152881.

98. Cristescu R, Lee J, Nebozhyn M, et al. Molecular analysis of gastric cancer identifies subtypes associated with distinct clinical outcomes. 2015;21(5):449-456.

99. Ma Y, Wu L, Liu C, Xu L, Li D, Li JC. The correlation of genetic instability of PINX1 gene to clinico-pathological features of gastric cancer in the Chinese population. *Journal of cancer research and clinical oncology.* 2009;135(3):431-437.

100. Ma C, Patel K, Singhi AD, et al. Programmed Death-Ligand 1 Expression Is Common in Gastric Cancer Associated With Epstein-Barr Virus or Microsatellite Instability. *The American journal of surgical pathology.* 2016;40(11):1496-1506.

101. Mizoshita T, Tsukamoto T, Cao X, et al. Microsatellite instability is linked to loss of hMLH1 expression in advanced gastric cancers: lack of a relationship with the histological type and phenotype. *Gastric cancer : official journal of the International Gastric Cancer Association and the Japanese Gastric Cancer Association.* 2005;8(3):164-172.

102. Moehler M, Dvorkin M, Boku N, et al. Phase III Trial of Avelumab Maintenance After First-Line Induction Chemotherapy Versus Continuation of Chemotherapy in Patients With Gastric Cancers: Results From JAVELIN Gastric 100. *J Clin Oncol.* 2020:JCO2000892.

103. Oh N, Kim H, Kim KM, et al. Microsatellite Instability and Effectiveness of Adjuvant Treatment in pT1N1 Gastric Cancer: A Multicohort Study. *Annals of surgical oncology.* 2021;28(13):8908-8915.

104. Oki E, Kakeji Y, Zhao Y, et al. Chemosensitivity and survival in gastric cancer patients with microsatellite instability. *Annals of surgical oncology.* 2009;16(9):2510-2515.

105. Oki E, Zhao Y, Yoshida R, et al. Checkpoint with forkhead-associated and ring finger promoter hypermethylation correlates with microsatellite instability in gastric cancer. *World journal of gastroenterology.* 2009;15(20):2520-2525.

106. Sakurai M, Zhao Y, Oki E, Kakeji Y, Oda S, Maehara Y. High-resolution fluorescent analysis of microsatellite instability in gastric cancer. *European journal of gastroenterology & hepatology.* 2007;19(8):701-709.

107. Ottini L, Palli D, Falchetti M, et al. Microsatellite instability in gastric cancer is associated with tumor location and family history in a high-risk population from Tuscany. *Cancer research.* 1997;57(20):4523-4529.

108. Palli D, Russo A, Ottini L, et al. Red meat, family history, and increased risk of gastric cancer with microsatellite instability. *Cancer research.* 2001;61(14):5415-5419.

109. Park SY, Kook MC, Kim YW, Cho NY, Kim TY, Kang GH. Mixed-type gastric cancer and its association with high-frequency CpG island hypermethylation. *Virchows Archiv : an international journal of pathology.* 2010;456(6):625-633.

110. Park J, Yoo HM, Jang W, et al. Distribution of somatic mutations of cancer-related genes according to microsatellite instability status in Korean gastric cancer. *Medicine.* 2017;96(25):e7224.

111. Pascua I, Fernández-Marcelo T, Sánchez-Pernaute A, et al. Prognostic value of telomere function in gastric cancers with and without microsatellite instability. *European journal of gastroenterology & hepatology.* 2015;27(2):162-169.

112. Pereira MA, Ramos M, Faraj SF, et al. Clinicopathological and prognostic features of Epstein-Barr virus infection, microsatellite instability, and PD-L1 expression in gastric cancer. *Journal of surgical oncology.* 2018;117(5):829-839.

113. Pietrantonio F, Miceli R, Raimondi A, et al. Individual Patient Data Meta-Analysis of the Value of Microsatellite Instability As a Biomarker in Gastric Cancer. *Journal of clinical oncology : official journal of the American Society of Clinical Oncology.* 2019;37(35):3392-3400.

114. Pinto M, Oliveira C, Machado JC, et al. MSI-L gastric carcinomas share the hMLH1 methylation status of MSI-H carcinomas but not their clinicopathological profile. *Laboratory investigation; a journal of technical methods and pathology.* 2000;80(12):1915-1923.

115. dos Santos NR, Seruca R, Constância M, Seixas M, Sobrinho-Simões M. Microsatellite instability at multiple loci in gastric carcinoma: clinicopathologic implications and prognosis. *Gastroenterology.* 1996;110(1):38-44.

116. Polom K, Böger C, Smyth E, et al. Synchronous metastatic gastric cancer-molecular background and clinical implications with special attention to mismatch repair deficiency. *European journal of surgical oncology : the journal of the European Society of Surgical Oncology and the British Association of Surgical Oncology.* 2018;44(5):626-631.

117. Polom K, Das K, Marrelli D, et al. KRAS Mutation in Gastric Cancer and Prognostication Associated with Microsatellite Instability Status. *Pathology oncology research : POR.* 2019;25(1):333-340.

118. Polom K, Marrelli D, Pascale V, et al. High-risk and low-risk gastric cancer areas in Italy and its association with microsatellite instability. *Journal of cancer research and clinical oncology.* 2016;142(8):1817-1824.

119. Pedrazzani C, Corso G, Velho S, et al. Evidence of tumor microsatellite instability in gastric cancer with familial aggregation. *Familial cancer.* 2009;8(3):215-220.

120. Marrelli D, Polom K, Pascale V, et al. Strong Prognostic Value of Microsatellite Instability in Intestinal Type Non-cardia Gastric Cancer. *Annals of surgical oncology.* 2016;23(3):943-950.

121. Corso G, Pedrazzani C, Marrelli D, Pascale V, Pinto E, Roviello F. Correlation of microsatellite instability at multiple loci with long-term survival in advanced gastric carcinoma. *Archives of surgery (Chicago, Ill : 1960).* 2009;144(8):722-727.

122. Corso G, Velho S, Paredes J, et al. Oncogenic mutations in gastric cancer with microsatellite instability. *European journal of cancer (Oxford, England : 1990).* 2011;47(3):443-451.

123. Pretzsch E, Bösch F, Todorova R, et al. Molecular subtyping of gastric cancer according to ACRG using immunohistochemistry - Correlation with clinical parameters. *Pathology, research and practice.* 2022;231:153797.

124. Quaas A, Rehkaemper J, Rueschoff J, et al. Occurrence of High Microsatellite-Instability/Mismatch Repair Deficiency in Nearly 2,000 Human Adenocarcinomas of the Gastrointestinal Tract, Pancreas, and Bile Ducts: A Study From a Large German Comprehensive Cancer Center. *Frontiers in oncology.* 2021;11:569475.

125. Ramos M, Pereira MA, de Mello ES, et al. Gastric cancer molecular classification based on immunohistochemistry and in situ hybridization: Analysis in western patients after curative-intent surgery. *World journal of clinical oncology.* 2021;12(8):688-701.

126. Renault B, Calistri D, Buonsanti G, Nanni O, Amadori D, Ranzani GN. Microsatellite instability and mutations of p53 and TGF-beta RII genes in gastric cancer. *Human genetics.* 1996;98(5):601-607.

127. Rugge M, Bersani G, Bertorelle R, et al. Microsatellite instability and gastric non-invasive neoplasia in a high risk population in Cesena, Italy. *Journal of clinical pathology.* 2005;58(8):805-810.

128. Schlintl V, Huemer F, Rinnerthaler G, et al. Checkpoint inhibitors in metastatic gastric and GEJ cancer: a multi-institutional retrospective analysis of real-world data in a Western cohort. *BMC cancer.* 2022;22(1):51.

129. Schneider BG, Bravo JC, Roa JC, et al. Microsatellite instability, prognosis and metastasis in gastric cancers from a low-risk population. *Int J Cancer.* 2000;89(5):444-452.

130. Seo HM, Chang YS, Joo SH, et al. Clinicopathologic characteristics and outcomes of gastric cancers with the MSI-H phenotype. *Journal of surgical oncology.* 2009;99(3):143-147.

131. Sepulveda AR, Santos AC, Yamaoka Y, et al. Marked differences in the frequency of microsatellite instability in gastric cancer from different countries. *The American journal of gastroenterology.* 1999;94(10):3034-3038.

132. Seruca R, Santos NR, David L, et al. Sporadic gastric carcinomas with microsatellite instability display a particular clinicopathologic profile. *Int J Cancer.* 1995;64(1):32-36.

133. Shen H, Zhong M, Wang W, et al. EBV infection and MSI status significantly influence the clinical outcomes of gastric cancer patients. *Clinica chimica acta; international journal of clinical chemistry.* 2017;471:216-221.

134. Shirai K, Ohmiya N, Taguchi A, et al. Interleukin-8 gene polymorphism associated with susceptibility to non-cardia gastric carcinoma with microsatellite instability. *Journal of gastroenterology and hepatology.* 2006;21(7):1129-1135.

135. Shitara K, Ajani JA, Moehler M, et al. Nivolumab plus chemotherapy or ipilimumab in gastro-oesophageal cancer. *Nature.* 2022;603(7903):942-948.

136. Janjigian YY, Shitara K, Moehler M, et al. First-line nivolumab plus chemotherapy versus chemotherapy alone for advanced gastric, gastro-oesophageal junction, and oesophageal adenocarcinoma (CheckMate 649): a randomised, open-label, phase 3 trial. *Lancet (London, England).* 2021;398(10294):27-40.

137. Stanek L, Gurlich R, Musil Z, Havluj L, Whitley A. Monitoring EBV infection, MSI, PDL-1 expression, Her-2/neu amplification as a biomarker for PD-1 inhibition in gastric cancer. *Bratislavske lekarske listy.* 2022;123(2):83-86.

138. Sugimoto R, Endo M, Osakabe M, et al. Immunohistochemical Analysis of Mismatch Repair Gene Proteins in Early Gastric Cancer Based on Microsatellite Status. *Digestion.* 2021;102(5):691-700.

139. Sugai T, Eizuka M, Arakawa N, et al. Molecular profiling and comprehensive genome-wide analysis of somatic copy number alterations in gastric intramucosal neoplasias based on microsatellite status. *Gastric cancer : official journal of the International Gastric Cancer Association and the Japanese Gastric Cancer Association.* 2018;21(5):765-775.

140. Sugimoto R, Sugai T, Habano W, et al. Clinicopathological and molecular alterations in early gastric cancers with the microsatellite instability-high phenotype. *Int J Cancer.* 2016;138(7):1689-1697.

141. Takahashi H, Endo T, Yamashita K, et al. Mucin phenotype and microsatellite instability in early multiple gastric cancers. *Int J Cancer.* 2002;100(4):419-424.

142. Tanabe H, Mizukami Y, Takei H, et al. Clinicopathological characteristics of Epstein-Barr virus and microsatellite instability subtypes of early gastric neoplasms classified by the Japanese and the World Health Organization criteria. *The journal of pathology Clinical research.* 2021;7(4):397-409.

143. Theuer CP, Campbell BS, Peel DJ, et al. Microsatellite instability in Japanese vs European American patients with gastric cancer. *Archives of surgery (Chicago, Ill : 1960).* 2002;137(8):960-965; discussion 965-966.

144. Vauhkonen M, Vauhkonen H, Sajantila A, Sipponen P. Differences in genomic instability between intestinal- and diffuse-type gastric cancer. *Gastric cancer : official journal of the International Gastric Cancer Association and the Japanese Gastric Cancer Association.* 2005;8(4):238-244.

145. Velho S, Oliveira C, Ferreira A, et al. The prevalence of PIK3CA mutations in gastric and colon cancer. *European journal of cancer (Oxford, England : 1990).* 2005;41(11):1649-1654.

146. Wang YL, Gong Y, Lv Z, Li L, Yuan Y. Expression of PD1/PDL1 in gastric cancer at different microsatellite status and its correlation with infiltrating immune cells in the tumor microenvironment. *Journal of Cancer.* 2021;12(6):1698-1707.

147. Wu M, Semba S, Oue N, Ikehara N, Yasui W, Yokozaki H. BRAF/K-ras mutation, microsatellite instability, and promoter hypermethylation of hMLH1/MGMT in human gastric carcinomas. *Gastric cancer : official journal of the International Gastric Cancer Association and the Japanese Gastric Cancer Association.* 2004;7(4):246-253.

148. Wu CW, Chen MH, Huang KH, et al. The clinicopathological characteristics and genetic alterations between younger and older gastric cancer patients with curative surgery. *Aging.* 2020;12(18):18137-18150.

149. Fang WL, Chang SC, Lan YT, et al. Microsatellite instability is associated with a better prognosis for gastric cancer patients after curative surgery. *World journal of surgery.* 2012;36(9):2131-2138.

150. Xiao YP, Wu DY, Xu L, Xin Y. Loss of heterozygosity and microsatellite instabilities of fragile histidine triad gene in gastric carcinoma. *World journal of gastroenterology.* 2006;12(23):3766-3769.

151. Yamada T, Koyama T, Ohwada S, et al. Frameshift mutations in the MBD4/MED1 gene in primary gastric cancer with high-frequency microsatellite instability. *Cancer letters.* 2002;181(1):115-120.

152. Yamashita K, Arimura Y, Saito M, et al. Gastric cancers with microsatellite instability sharing clinical features, chemoresistance and germline MSH6 variants. *Clinical journal of gastroenterology.* 2013;6(2):122-126.

153. Yamazaki K, Tajima Y, Makino R, et al. Tumor differentiation phenotype in gastric differentiated-type tumors and its relation to tumor invasion and genetic alterations. *World journal of gastroenterology.* 2006;12(24):3803-3809.

154. Yoshida T, Ogura G, Tanabe M, et al. Clinicopathological features of PD-L1 protein expression, EBV positivity, and MSI status in patients with advanced gastric and esophagogastric junction adenocarcinoma in Japan. *Cancer biology & therapy.* 2022;23(1):191-200.

155. Yu P, Wang Y, Yu Y, et al. Deep Targeted Sequencing and Its Potential Implication for Cancer Therapy in Chinese Patients with Gastric Adenocarcinoma. *The oncologist.* 2021;26(5):e756-e768.

156. Yu HY, Li CP, Huang YH, et al. Microsatellite Instability, Epstein-Barr Virus, and Programmed Cell Death Ligand 1 as Predictive Markers for Immunotherapy in Gastric Cancer. *Cancers (Basel).* 2022;14(1).

157. Yuza K, Nagahashi M, Ichikawa H, et al. Activin a Receptor Type 2A Mutation Affects the Tumor Biology of Microsatellite Instability-High Gastric Cancer. *Journal of gastrointestinal surgery : official journal of the Society for Surgery of the Alimentary Tract.* 2021;25(9):2231-2241.

158. Zaky AH, Watari J, Tanabe H, et al. Clinicopathologic implications of genetic instability in intestinal-type gastric cancer and intestinal metaplasia as a precancerous lesion: proof of field cancerization in the stomach. *American journal of clinical pathology.* 2008;129(4):613-621.

159. Zhang Q, Wang L, Ni S, et al. Clinicopathological features and prognostic value of mismatch repair protein deficiency in gastric cancer. *International journal of clinical and experimental pathology.* 2018;11(5):2579-2587.

160. Zhang L, Wang Y, Li Z, et al. Clinicopathological features of tumor mutation burden, Epstein-Barr virus infection, microsatellite instability and PD-L1 status in Chinese patients with gastric cancer. *Diagnostic pathology.* 2021;16(1):38.

161. Wang Z, Wang X, Xu Y, et al. Mutations of PI3K-AKT-mTOR pathway as predictors for immune cell infiltration and immunotherapy efficacy in dMMR/MSI-H gastric adenocarcinoma. *BMC medicine.* 2022;20(1):133.

162. Zhang M, Qi C, Wang Z, et al. Molecular characterization of ctDNA from Chinese patients with advanced gastric adenocarcinoma reveals actionable alterations for targeted and immune therapy. *Journal of molecular medicine (Berlin, Germany).* 2021;99(9):1311-1321.

163. Zhao W, Chan TL, Chu KM, et al. Mutations of BRAF and KRAS in gastric cancer and their association with microsatellite instability. *Int J Cancer.* 2004;108(1):167-169.

164. Leung SY, Yuen ST, Chung LP, et al. Microsatellite instability, Epstein-Barr virus, mutation of type II transforming growth factor beta receptor and BAX in gastric carcinomas in Hong Kong Chinese. *British journal of cancer.* 1999;79(3-4):582-588.

165. Zhao Y, Zheng ZC, Luo YH, et al. Low-frequency microsatellite instability in genomic di-nucleotide sequences correlates with lymphatic invasion and poor prognosis in gastric cancer. *Cell biochemistry and biophysics.* 2015;71(1):235-241.
